# The Woody-Preferential Gene *EgMYB88* Regulates the Biosynthesis of Phenylpropanoid-Derived Compounds in Wood

**DOI:** 10.3389/fpls.2016.01422

**Published:** 2016-09-22

**Authors:** Marçal Soler, Anna Plasencia, Jorge Lepikson-Neto, Eduardo L. O. Camargo, Annabelle Dupas, Nathalie Ladouce, Edouard Pesquet, Fabien Mounet, Romain Larbat, Jacqueline Grima-Pettenati

**Affiliations:** ^1^Laboratoire de Recherche en Sciences Végétales, Centre National de la Recherche Scientifique, Université de Toulouse III, Paul SabatierToulouse, France; ^2^Department of Plant Physiology, Umeå UniversityUmeå, Sweden; ^3^“Agronomie et Environnement” Nancy-Colmar, Institut National de la Recherche Agronomique, Université de Lorraine UMR1121Vandœuvre-lès-Nancy, France

**Keywords:** vascular cambium, MYB transcription factors, phenylpropanoid metabolism, lignin, oligolignols, flavonoids, salicinoid phenolic glycosides, *Eucalyptus*

## Abstract

Comparative phylogenetic analyses of the R2R3-MYB transcription factor family revealed that five subgroups were preferentially found in woody species and were totally absent from Brassicaceae and monocots (Soler et al., 2015). Here, we analyzed one of these subgroups (WPS-I) for which no gene had been yet characterized. Most *Eucalyptus* members of WPS-I are preferentially expressed in the vascular cambium, the secondary meristem responsible for tree radial growth. We focused on *EgMYB88*, which is the most specifically and highly expressed in vascular tissues, and showed that it behaves as a transcriptional activator in yeast. Then, we functionally characterized *EgMYB88* in both transgenic *Arabidopsis* and poplar plants overexpressing either the native or the dominant repression form (fused to the Ethylene-responsive element binding factor-associated Amphiphilic Repression motif, EAR). The transgenic *Arabidopsis* lines had no phenotype whereas the poplar lines overexpressing *EgMYB88* exhibited a substantial increase in the levels of the flavonoid catechin and of some salicinoid phenolic glycosides (salicortin, salireposide, and tremulacin), in agreement with the increase of the transcript levels of landmark biosynthetic genes. A change in the lignin structure (increase in the syringyl vs. guaiacyl, S/G ratio) was also observed. Poplar lines overexpressing the *EgMYB88* dominant repression form did not show a strict opposite phenotype. The level of catechin was reduced, but the levels of the salicinoid phenolic glycosides and the S/G ratio remained unchanged. In addition, they showed a reduction in soluble oligolignols containing sinapyl *p*-hydroxybenzoate accompanied by a mild reduction of the insoluble lignin content. Altogether, these results suggest that *EgMYB88*, and more largely members of the WPS-I group, could control in cambium and in the first layers of differentiating xylem the biosynthesis of some phenylpropanoid-derived secondary metabolites including lignin.

## Introduction

Eucalypts have extremely high adaptive potential and a considerable capacity to produce lignocellulosic biomass, explaining why this genus is the most planted hardwood worldwide for many industrial uses (Myburg et al., 2014). As tree species, eucalypts are long-living organisms characterized by a massive secondary growth produced by the activity of a secondary meristem, called the vascular cambium. Although essential for secondary growth, the vascular cambium is difficult to study because of its internal location and has received much less attention that the other plant meristems. During tree radial growth, the cambial cells divide and differentiate centripetally into xylem cells, characterized by thick secondary cell walls made of cellulose (40–50%), hemicelluloses (25%), and lignin (25–35%) (Plomion et al., 2001).

Lignin is a complex phenolic polymer which ensures essential functions for terrestrial plants providing mechanical support and facilitating the water transport. It is composed of mainly two units, syringyl (S) and guaiacyl (G), with also minor amounts of *p*-hydroxyphenyl (H) units, all produced by the monolignol-specific branch of the general phenylpropanoid metabolism. In addition to lignin monomers, the phenylpropanoid metabolism serves as a starting point for a vast array of other important compounds, such as flavonoids (flavonols, anthocyans, and proanthocyanidins), coumarins (Vogt, 2010), or phenolic glycosides (Babst et al., 2010). All these phenylpropanoid-derived secondary metabolites are crucial for plant survival, contributing to all aspects of plant responses toward biotic and abiotic stimuli (Vogt, 2010).

R2R3-MYB genes constitute one of the largest families of transcription factors in plants. They regulate many aspects of plant biology, such as primary and secondary metabolism, cell fate, developmental processes, and responses to biotic and abiotic stresses. Noteworthy, more than 30% of these genes characterized in *Arabidopsis* regulate different aspects of the phenylpropanoid metabolism, including the biosynthesis of lignin and flavonoids (Dubos et al., 2010). R2R3-MYB proteins are characterized by a highly conserved N-terminal DNA-binding domain (R2R3-MYB domain) and a highly variable C-terminal activation or repression domain. The combination of phylogenetic studies with the detection of conserved motives in the C-terminal region enabled to define 22 subgroups in *Arabidopsis* (Stracke et al., 2001) found to be quite well conserved in other species. Interestingly, genes within the same subgroup are thought to realize similar functions (Dubos et al., 2010).

Recently, the phylogenetic analysis of the R2R3-MYB members from *Eucalyptus grandis, Arabidopsis thaliana, Populus trichocarpa, Vitis vinifera*, and *Oryza sativa* allowed to identify five subgroups of R2R3-MYB proteins preferentially found in woody species, named as Woody Preferential Subgroups (WPS-I, II, III, IV, and V), which are totally absent in the basal lineages of the Bryophytes and Lycophytes, as well as in the more modern Monocot and Brassicaceae lineages (Soler et al., 2015). *E. grandis* genes from WPS-I, II, and III, are preferentially expressed in the cambial region, and given the close phylogenetic relationship with genes from subgroups involved in the regulation of the phenylpropanoid metabolism such as S4, S5, S6, S7, and SAtMYB5 (Dubos et al., 2010), it could be hypothesized that genes belonging to WPS-I, II, and III regulate the biosynthesis of some phenylpropanoid-derived compounds (Soler et al., 2015). Indeed, some genes belonging to WPS-II and III, but attributed earlier to subdivisions of S4 or S5, have already been shown to regulate the biosynthesis of flavonoids. For example, the two genes characterized from WPS-II (*VvMYBPA1* from grapevine and *DkMYB2* from persimmon) act as activators of the biosynthesis of proanthocyanidins and other phenylpropanoid-derived compounds (Bogs et al., 2007; Akagi et al., 2010), whereas the genes characterized from WPS-III (*FaMYB1* from *Fragaria* x *ananasa, FcMYB1* from *Fragaria chiloensis, VvMYBC2-L1*, and *VvMYBC2-L3* from grapevine, *PhMYB27* from petunia and PtMYB182 in poplar) act as repressors of the biosynthesis of these compounds (Aharoni et al., 2001; Salvatierra et al., 2013; Albert et al., 2014; Huang et al., 2014; Cavallini et al., 2015; Yoshida et al., 2015). These studies focused mostly on leaves, fruits, flowers, or *in vitro* cultivated hairy roots. None has investigated the function of these genes in cambium and/or in differentiating xylem, nor their effects over lignin content and composition. Moreover, no gene belonging to WPS-I has been characterized to date in any plant species.

With the aim to better understand the role of WPS-I R2R3 MYBs, we functionally characterized a representative member, *EgMYB88*, shown to be highly and preferentially expressed in the cambial region. We showed that it behaves as an autoactivator of *Gal4* in yeast. Given the considerable difficulty to obtain transgenic eucalypts, we decided to overexpress *EgMYB88* in both *Arabidopsis* and poplar, either as a native form or fused to an active repressor motif [Ethylene-responsive element binding factor-associated Amphiphilic Repression (EAR)] to transform it into a dominant repressor (Hiratsu et al., 2003). As could be expected from a gene absent in Brassicaceae, the *Arabidopsis* transgenic lines showed no phenotypic differences compared to controls. In contrast, poplar transgenic lines overexpressing *EgMYB88* showed a substantial increase in phenolic glycosides and flavonoids concomitant with a modification of the lignin structure (increase in the S/G ratio). Poplar lines overexpressing *EgMYB88-EAR* showed a significant reduction of flavonoids and soluble oligolignols accompanied by a reduction of the lignin content. Altogether, these results suggest that the main role of *EgMYB88* in the cambial region, and likely of its orthologs in poplar, is to control specific branches of the phenylpropanoid metabolism.

## Materials and methods

### Phylogenetic analysis

Sequences of MYB proteins from *E. grandis, P. trichocarpa, V. vinifera*, and *A. thaliana* from a phenylpropanoid metabolism-related super clade were obtained from Soler et al. (2015). It includes members of S4 (with AtMYB6 and AtMYB8), S5, S6, S7, S15, SAtMYB5, SAtMYB82, WPS-I, WPS-II, and WPS-III. For *P. trichocarpa*, we updated the protein sequences from the last genome version (3.0). Two gene models from version 2.2 were not included in the analysis: POPTR_0019s05200 was absent in version 3.0, whereas the new amino acid sequence prediction of POPTR_0018s08500 (Potri.018G049000) lacked part of the R3 MYB domain.

Amino acid sequences were aligned using MAFFT with the FFT-NS-i algorithm (Katoh et al., 2002) and used to construct a Neighbor-joining phylogenetic tree using Mega5 (Tamura et al., 2011) with 1000 bootstrap replicates. Sequences were compared using the complete deletion method and the evolutionary distances were computed with the Jones–Taylor–Thornton substitution model using the rate variation among sites with a gamma distribution of 1, as done by Soler et al. (2015). A distant R2R3-MYB gene involved in the lignin biosynthesis, AtMYB52 (Cassan-Wang et al., 2013) was also used to root the phylogenetic reconstruction.

### Gene cloning and vector construction

*EgMYB88* coding sequence was obtained from *Eucalyptus gunnii* (*Eg*) xylem cDNA using the primers CACCCATATGGAGAAATCATCAGCTGCAA and GAGCTCTCCTGATCTCTCATCACA with the Phusion taq (Finnzymes). *EgMYB88* sequence from *E. gunnii* (accession number KX470407) was highly similar to *EgrMYB88* (*Egr* indicates *E. grandis*) sequence (Eucgr.F04423.1), with just one nucleotide change at the R3 MYB domain which do not modify the amino acid sequence. Amplicon was then inserted in the pENTR D-TOPO vector (Invitrogen) and subsequently transferred into several destination vectors using LR clonase II (Invitrogen) following manufacturer's instructions. Destination vectors were pGBD-GTW (kindly provide by Dr Laurent Deslandes, LIPM, INRA, France) for yeast auto activation assays, pFAST-G02 (Shimada et al., 2010) for overexpression in *Arabidopsis*, pJCV53 (obtained from the Gateway Vectors facility from Ghent University, Belgium) for overexpression in poplar, and pH35SGEAR (kindly provided by Dr Taku Demura, NAIST, Nara, Japan) for dominant repression in *Arabidopsis* and poplar. Coding sequences of EgMYB1 and EgMYB2 (Goicoechea et al., 2005; Legay et al., 2007) were cloned into the pDONR207 vector (Invitrogen) and were transferred into the pGBD-GTW vector also using the LR clonase II.

### Plant material

*EgMYB88* cloned into the pFAST-G02, the pJCV53, or the pH35SGEAR vectors was inserted into *Agrobacterium tumefaciens* GV3101 (pMP90) using freeze and thaw method. Wild type Col-0 *Arabidopsis* plants were transformed using the floral dip method (Clough and Bent, 1998) with *A. tumefaciens* harboring *EgMYB88* into the pFAST-G02 and the pH35SGEAR vectors. As controls, we used wild type plants and plants transformed with the respective empty vectors. Transformed seeds from pFAST-G02 vector were screened using a fluorescent stereomicroscope with GFP filters as described in Shimada et al. (2010), whereas seedlings transformed with the pH35SGEAR vector were screened using MS ½ media supplemented with hygromcycin (20 μg/ml). More than 10 transformed independent lines without any visible phenotype as compared to controls were obtained for each construct and screened by conventional RetroTranscriptase-quantitative Polymerase Chain Reaction (RT-qPCR) to assess the level of transgene expression. For detailed characterization of their phenotypes, three lines were selected for each construct based on high transgene expression levels (Figure S1). Plants were grown in a growth chamber in short day conditions (9 h light and 15 h dark) to promote secondary growth. Sampling was done in plants from at least 8 weeks old, when the inflorescence stems were fully developed and the first siliques were clearly observed. The base of the inflorescence stem was kept in ethanol 80% for histochemistry analysis. The rest of the inflorescence stem without leaves and siliques (except the first 5 cm, which were discarded because the proportion of xylem tissue was very low) was immediately frozen in liquid nitrogen. Five plants samples were pooled for each independent line. They were subsequently milled to powder using a ball-mill (MM400, Retsch) with liquid nitrogen to keep them protected from degradation, and kept at −80°C for lignin quantification. Similarly, hypocotyls were harvested, pooled, frozen in liquid nitrogen, milled to powder and kept at −80°C for Pyrolysis-Gas Chromatography/Mass Spectrometry (Py-GC/MS) phenolic profiling.

Hybrid *Populus tremula* x *P. alba* (INRA clone 717-1-B4) was maintained on MS-1B media (Duchefa) supplemented with 2% sucrose, 0.5 mg/L of IAA (indole-3-acetic acid, Duchefa), and 0.5 mg/L of IBA (indole-3-butyric acid, Sigma). Hybrid poplar was transformed with *A. tumefaciens* harboring *EgMYB88* into the pJCV53 and the pH35SGEAR vectors following the protocol described by Gallardo et al. (1999). As controls we used both wild type plants and plants transformed with the empty vectors. Selection of transformants was performed using kanamycin (50 μg/ml) for the pJCV53 and hygromycin (20 μg/ml) for pH35GEAR transformed plantlets. Several transformed plantlets were obtained for each construct without any visible phenotype as compared to controls, and screened by conventional RT-qPCR to assess the level of transgene expression. For detailed characterization of their phenotypes, three lines were selected for each construct according to transgene expression levels (Figure S2); however, for plants overexpressing *EgMYB88* into the pJCV53 vector we did not chose the lines with the highest transgene expression as they did not multiply well *in vitro*. Selected plants were then acclimated for 3 weeks in a growth chamber in long day conditions (16 h light and 8 h dark) and then transferred into a greenhouse for 2 months. Sampling was done taking a 5 cm portion of the base of the stem and kept it in ethanol 80% for histochemistry analysis. The subsequent 5 cm portion of the base of the stem, taken for transcriptomics, as well as the rest of the stem (except the first 12 internodes, discarded because the proportion of xylem was low), taken for biochemistry, were immediately debarked, frozen in liquid nitrogen, milled to powder and kept at −80 °C for transcriptomic and biochemical analyses.

### Yeast autoactivation tests

Yeast cells, strain Y2HGold (Clontech), were transformed with the plasmid pGBD-GTW using the Yeast Transformation System 2 (Clontech) following manufacturer's instructions and plating them on a Synthetically Defined medium without Tryptophan (SD-Trp). Plasmid pGBD-GTW contains a gene which confers the ability to grow on media without Tryptophan. Transformed yeast cells were resuspended in NaCl 0.9% solution until OD_600_ of 2 and then plated in different selective media. After 3 days incubation at 30°C, growing of yeast cells was visible.

### Transcriptomic analysis

Total RNA was extracted from *Arabidopsis* leaves using the protocol for “vegetative tissues” from Oñate-Sánchez and Vicente-Carbajosa (2008) and from poplar leaves and debarked stems using the protocol from Southerton et al. (1998). RNA was further digested using TurboDNase (Ambion) to remove residual traces of contaminating DNA. One Microgram of RNA was retrotranscribed into cDNA using the High Capacity RNA to cDNA kit (Applied Biosystems) and diluted 5 times before use as template in conventional or microfluidic RT-qPCR.

Conventional RT-qPCR was performed on cDNA from *Arabidopsis* and poplar leaves to assess the level of transgene expression in technical triplicates using ABI 7900HT real-time PCR system (Applied Biosystems) with the Power SYBR Green PCR Master Mix (Applied Biosystems). PCR conditions were 95 °C for 10 min, followed by 40 cycles of 95°C for 15 s and 60°C for 1 min. After amplification, a dissociation step was performed to confirm the presence of a simple amplicon. Transcript abundance was calculated using the 2^−ΔΔCt^ method, with the housekeeping gene *actin2* (*ACT2*, Legay et al., 2010) for *Arabidopsis* and the housekeeping gene *cell division cycle2* (*CDC2*, Legay et al., 2010) for poplar to normalize data. Control samples were used to standardize results.

Microfluidic RT-qPCR was performed on cDNA from poplar debarked stems to assess the levels of phenylpropanoid metabolism genes using the Biomark® 96.96 Dynamic Array platform (Fluidigm) as described in Cassan-Wang et al. (2012). After amplification, a dissociation step was performed to confirm the presence of a simple amplicon. Transcript abundance was calculated using the 2^−ΔΔCt^ method, with the geometric mean of five validated housekeeping genes to normalize the results [*Ubiquitin* (*UBQ*), *CDC2* (Legay et al., 2010), *HK1, HK3*, and *HK11* (Sixto et al., 2016)]. Control samples were used to standardize the results. Finally, data was used in MeV (Saeed et al., 2003) to generate a heatmap.

Genes analyzed by microfluidic RT-qPCR were selected according to their importance in the phenylpropanoid metabolism or in cambium activity and differentiation, based on literature search (Ranocha et al., 2002; Shi et al., 2010; Berthet et al., 2011; Sun et al., 2011; Huang et al., 2012; Vanholme et al., 2013; Zhu et al., 2013; Liu et al., 2014a,b; Chedgy et al., 2015; Etchells et al., 2015; Yoshida et al., 2015). Primer sequences were obtained from literature whenever possible or designed either using Quantprime (Arvidsson et al., 2008) or Primer3 (Rozen and Skaletsky, 2000). Primer sequences for RT-qPCR are detailed in Table S1.

### Histological analysis

*Arabidopsis* and poplar stems were cut into 90 μm-thick sections using a vibratome (VT 100S, Leica). Due to the strong lignification of secondary xylem of poplar stems, vibratome was equipped with a Sapphire blade (Delaware Diamond Knives). Lignified secondary cell walls were stained with phloroglucinol-HCl reagent (VWR) and observed immediately under a bright-field inverted microscope (DM IRBE, Leica) equipped with a CDD color camera (DFC300 FX, Leica).

### Biochemical analysis

Milled samples from *Arabidopsis* and poplar stems were lyophilized and then subjected to exhaustive extraction with water, then ethanol, ethanol/toluene (50/50) and acetone in a Soxhlet apparatus. Recovered extractive-free samples were dried at 60°C overnight. Lignin content was evaluated in *Arabidopsis* using the acetyl bromide lignin method on 10 mg samples as described by Soler et al. (2016). Klason procedure was performed as previously described (Méchin et al., 2014) to quantify the acid-insoluble lignin on 50 mg of poplar samples.

The poplar lignin structure was analyzed using the thioacidolysis method on 10 mg of extractive-free sample as described by Méchin et al. (2014). Briefly, we used 1 ml of thioacidolysis reagent per mg of sample (for 100 ml of solution: 2.5 ml of boron trifluoride, 10 ml of ethanethiol and 0.2 ml of tetracosane 1.25 mg/ml, up to 100 ml dioxane). An aliquot of this solution (5 μl) was trimethylsilylated with 100 μl of N,O-bis(trimethylsilyl)trifluoroacetamide and 10 μl of pyridine for 30 min at 60°C. The trimethylsilylated samples were injected in a Gas Chromatography/Mass Spectrometry (GC-MS) (TSQ Quantu, Thermo Scientific) fitted with an autosampler, a splitless injector (280°C), and a iontrap mass spectrometer operating in the electronic impact mode with a source at 220°C, an interface at 280°C, and a 50–650 m/z scanning range. The column was a ZB5-MSi (Phénomenex) operated in the temperature program mode (from 45 to 180°C at +30°C/min, then 180 to 260°C at +3°C/min) with helium carrier gas at 1.5 ml/min flow rate.

Pyrolysis–Gas Chromatography/Mass Spectrometry (Py-GC/MS) analysis were performed with 80 μg of lyophilized powder per injection using non-extracted *Arabidopsis* hypocotyls. Powder was applied to a pyrolyzer equipped with an auto sampler (PY-2020iD and AS-1020E, Frontier Lab) connected to a GC/MS (7890A/5975C, Agilent). Pyrolysate separation and analysis, including peak detection, integration, normalization, and identification was done according to Gerber et al. (2012).

### Metabolite profiling

A total of 30 mg of lyophilized powder from poplar stems was extracted with 1 mL methanol 70% blended for 1 min using an Ultra-thurrax and then centrifuged at 10,000 g for 10 min. The supernatant was transferred to a new tube and one additional mL was added to the pellet, vortexed and let for 2 h at room temperature. The mixture was centrifuged again at 10,000 g for 10 min and the recovered supernatant was mixed with the first one. The 2 mL solution was evaporated in a speed-vacuum until dryness. Then, dried pellet was dissolved in 500 μL methanol 70% and passed through 0.22 μm filter.

Extracts (1 μL) were analyzed on Ultra-High Performance Liquid Chromatography (U-HPLC) system (Shimadzu) equipped with a photoDiode Array Detector (DAD) and a mass spectrometer. Sample was separated on a C18 kinetex (100 × 2.1 mm) column (Phenomenex). The mobile phase consisted in 0.1% formic acid in ultra-pure water (solvent A) and 0.1% formic acid in methanol (solvent B).

The molecules were eluted through a gradient elution from 1 to 99% B for 13 min with a flow rate of 400 μL/min and then 3 min in 99% B. The column was then re-equilibrated to 1% B prior to the next run. Mass spectrometry analysis was carried out in ESI negative mode. Quantification was performed by measuring the area under each peak at 280, 320, or 350 nm, depending on the lambda max of each molecule. Total metabolic signal was performed at 280 nm. Due to co-eluting signal at 280 nm, catechin quantification was realized by measuring the area under peak at *m/z* 289, corresponding to the [M-H]^−^ of the molecule. Experimental exact masses and MS fragments were compared to metabolomics data banks (Respect: http://spectra.psc.riken.jp/; Mass Bank: http://massbank.jp/, DNP: http://dnp.chemnetbase.com/) and data available in the literature in order to identify the nature of the metabolites.

## Results

### The WPS-I belonging gene *EgrMYB88* is highly and preferentially expressed in the cambial region

**Figure 1:**
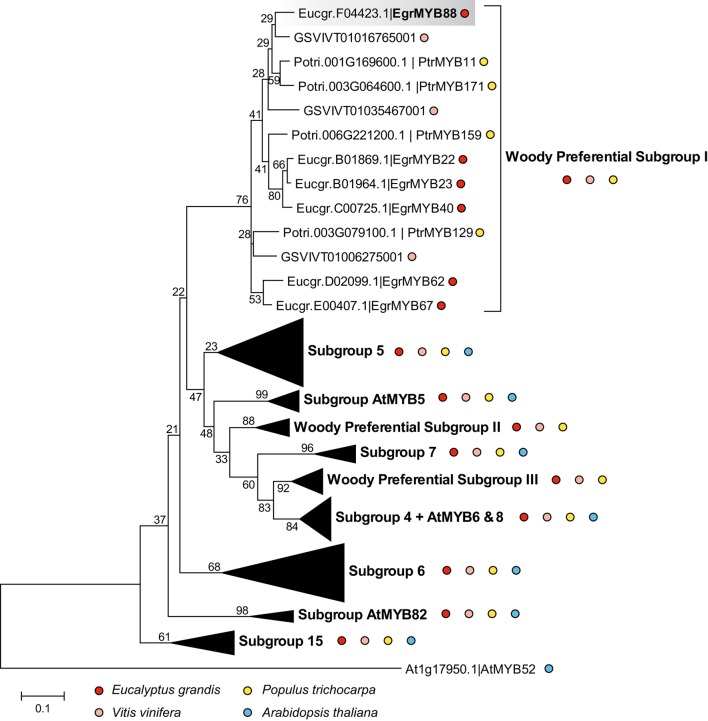
**Neighbor-Joining phylogenetic tree constructed with R2R3-MYB members of phenylpropanoid metabolism-related super clade**. The proteins included belong to the subgroups S4, S5, S6, S7, S15, SAtMYB5, SAtMYB82, WPS-I, WPS-II, and WPS-III from *E. grandis* (red dots), *V. vinifera* (pink dots), *P. trichocarpa* (yellow dots), and *A. thaliana* (blue dots). The distant protein AtMYB52 (Cassan-Wang et al., 2013), involved in the lignin biosynthesis, was used to root the tree. The WPS-I is expanded to show the different members from *E. grandis, P. trichocarpa* and *V. vinifera*, and the absence of genes from *Arabidopsis*. EgrMYB88 is highlighted with a gray background. Bootstrap values are shown on the internodes.

Based on the phylogeny of the whole R2R3-MYB family in *E. grandis* (Soler et al., 2015), we selected the genes from the WPS-I and from closely related subgroups (S4, S5, S6, S7, SAtMYB5, SAtMYB82, WPS-II, and III) that constitute a phenylpropanoid metabolism-related super-clade. We reconstructed a phylogenetic sub-tree using sequences of *E. grandis, A. thaliana, P. trichocarpa*, and *V. vinifera* (Figure 1). WPS-I is supported by a high bootstrap value and is clearly separated from the other subgroups. It contains six genes from *E. grandis*, four from poplar, three from grapevine, but none from *Arabidopsis*.

No segmental, whole genome or tandem duplications were previously detected for any of the WPS-I genes in the *E. grandis* genome (Soler et al., 2015). However, giving the extremely high sequence conservation (98% similarity at the protein level) between EgrMYB22 and EgrMYB23, the corresponding genes likely derived from a relatively recent duplication event further supported by their close location on the same chromosome (Figure S3, Table S2). All the other members of the WPS-I subgroup are located on different chromosomes. The EgrMYB88 protein sequence is the most divergent from the other *Eucalyptus* WPS-I members (Table S2). It has two putative orthologs in *P. trichocarpa*, PrtMYB11 and PtrMYB171 (Figure 1, Table S2).

**Figure 2:**
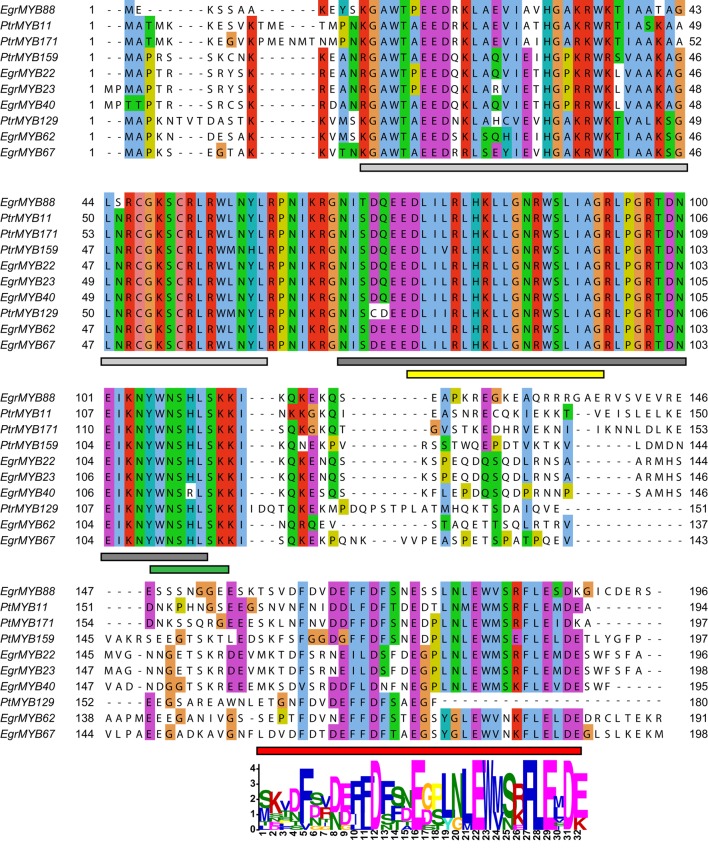
**Amino acid sequence alignment of the ***E. grandis*** and ***P. trichocarpa*** genes belonging to the WPS-I**. Amino acid are colored according to their similarity degree using the ClustalX option of Jalview (Waterhouse et al., 2009; http://www.jalview.org/help/html/colourSchemes/clustal.html). Light gray and dark gray rectangles at the bottom of the alignment indicate the R2 and the R3 MYB domains, respectively. Yellow rectangle indicates the bHLH interaction motif described by Zimmermann et al. (2004). Green rectangle indicates the region that, at the mRNA level, is targeted by the miR828 (Xia et al., 2012). Red rectangle indicates the position of the conserved C-terminal motif, whose sequence obtained using the MEME Suite (Bailey et al., 2009) is indicated below.

The six *E. grandis* and the four *P. trichocarpa* proteins exhibit high sequence homology in the R2R3-MYB domain, and also a conserved motif in the C-terminal region (Figure 2). This motif specific of the subgroup suggests similar function of the WPS-I genes. Moreover, as found for many other R2R3-MYB genes, a predicted motif for interaction with basic Helix-Loop-Helix (bHLH) proteins was also detected [(DE)Lx_2_(RK)x_3_Lx_6_Lx_3_R; Figure 2] (Zimmermann et al., 2004), which indicates that the function of these genes may be modulated by protein-protein interactions.

**Figure 3:**
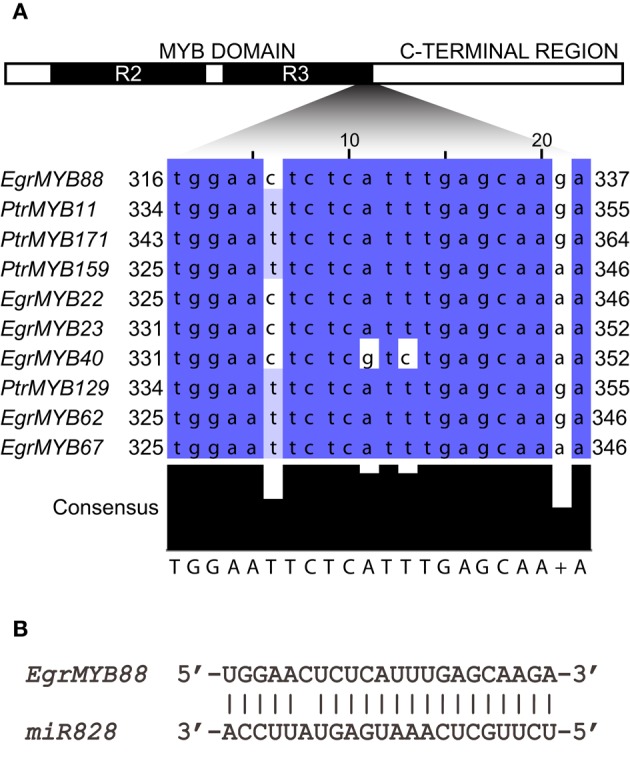
**Sequence region targeted by miR828 among the ***E. grandis*** and ***P. trichocarpa*** members of WPS-I. (A)** Gene nucleotide sequence alignment colored according to their degree of identity percentage using Jalview (Waterhouse et al., 2009; http://www.jalview.org/help/html/colourSchemes/pid.html). **(B)** Alignment between the *EgMYB88* mRNA and the miR828 showing one single mismatch among 22 nucleotides. Sequence targeted by miR828 is based on Xia et al. (2012).

Because WPS-I closely related subgroups such as S6 and S7 involved in flavonoids biosynthesis were shown to be post-transcriptionally repressed by microRNAs, (mostly miR828 and/or miR858; Xia et al., 2012), we examined if the genes of WPS-I could also be targets of these microRNAs. By performing an *in silico* search, we found that the miRNA828 recognition sequence located at the end of the R2R3-MYB domain was highly conserved (Figure 3) in all the WPS-I genes from *Eucalyptus* and poplar with the exception of *EgrMYB40*. No cleavage site for miR858 was found in any of these genes (data not shown). These results are in agreement with a *Eucalyptus* degradome analysis showing that *EgrMYB22, 23, 62, 67*, and *88*, but no *EgrMYB40*, were cleaved by miR828 (Carocha and Paiva, personal communication).

**Figure 4:**
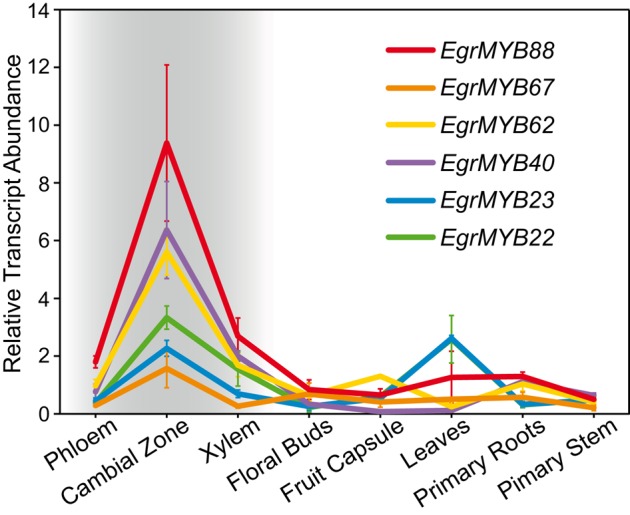
**Transcript abundance data of the different ***Eucalyptus*** genes from WPS-I**. Expression data (means ± standard deviation) in different organs and tissues from *Eucalyptus* was extracted from Soler et al. (2015). Vascular tissues are highlighted with a gray background. *EgrMYB88* is the gene with the highest expression in vascular tissues.

We then analyzed the transcript abundance of the genes from WPS-I in different *Eucalyptu*s organs and tissues previously described in Soler et al. (2015). All of them showed the highest expression in the cambial region although some were also expressed in other tissues/organs (Figure 4). *EgrMYB22*, for instance was also highly expressed in leaves. *EgrMYB88* was preferentially expressed in the vascular tissues (xylem and phloem) and exhibited the highest expression in the cambial region. The expression of its orthologs in *P. tremula* was obtained from Popgenie database (Sjödin et al., 2009) based on the RNAseq survey performed by Sundell et al. (2015). Similarly to *EgMYB88, PtMYB11*, and *PtMYB171* were highly expressed in wood, but exhibited distinct expression profiles in other non-woody tissues (Figure S4). Because of the importance of the vascular cambium and differentiating xylem in the secondary growth characteristic of woody plants, we decided to investigate the function of *EgrMYB88* by cloning its coding sequence from *E. gunnii* growing in Southwest France (*EgMYB88*). Nucleotide coding sequence is nearly identical between *EgrMYB88* and *EgMYB88*, with just a base change at the R3 MYB domain that do not modify the amino acid sequence.

### EgMYB88 behaves as a transcriptional activator in yeast

**Figure 5:**
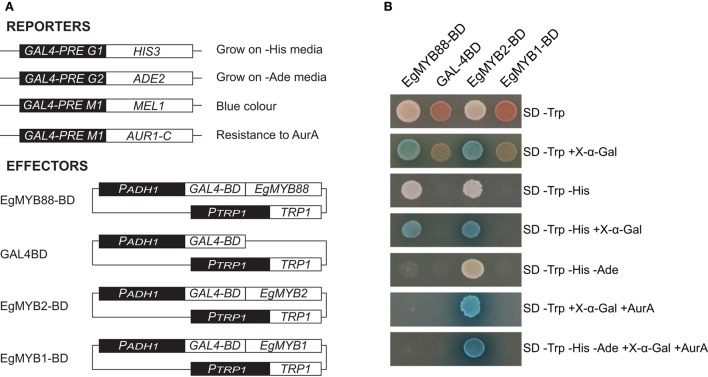
**Yeast auto-activation tests of EgMYB88 fused to the DNA binding domain of Gal4. (A)** Reporter and effector constructs used in the assay. The reporter constructs consists of the *cis*-regulatory elements to which Gal4 binds before the four reporter genes tested. The effector constructs consist of fusion proteins with the Gal4 binding domain (BD) at the N-terminal end. As controls, we tested the empty vector as well as two well-known *Eucalyptus* genes, one acting as an activator (EgMYB2, Goicoechea et al., 2005) and the other as a repressor (EgMYB1, Legay et al., 2007). **(B)** Growth of yeast cells in different selective media. Growth in auxotrophic media (SD) without Tryptophan (Trp) indicates the presence of the effector plasmid. Growth in the absence of Histidine (His) or Adenine (Ade) are indicative of activation of HIS3 and ADE2 reporter genes. Growth in presence of the antibiotic Aureobasidin A (AurA) indicates the activation of AUR1-C, which confers resistance to this antibiotic. Blue color represent the activation of MEL1 reporter gene, able to produce a blue precipitate in presence of 5-bromo-4-chloro-3-indolyl alpha-D-galactopyranoside (X-α-Gal).

To determine if EgMYB88 is a transcriptional activator, we fused it to the Gal4 binding domain and investigated its capacity to activate the transcription of four reporter genes in yeast (Figure 5A). EgMYB88 was able to activate the reporter gene *HIS3* and *MEL1*, as revealed by the ability of the yeast transformed with *EgMYB88* to grow on selective media lacking Histidine and to metabolize X-α-Gal (5-bromo-4-chloro-3-indolyl alpha-D-galactopyranoside) producing blue colonies (Figure 5B). EgMYB88 was, however, unable to induce the reporter gene *ADE2* and *AUR1C*, since the yeast transformed with *EgMYB88* did not grow on media lacking both Adenine and Histidine, or containing the antibiotic Aureobasidin A in addition to X-α-Gal. Thus, EgMYB88 behaves as an activator in yeast, but its activity seems weaker than that of EgMYB2, a known transcriptional activator (Goicoechea et al., 2005), which was able to activate the four reporter genes (Figure 5B). In contrast, EgMYB1, a known transcriptional repressor (Legay et al., 2007; Soler et al., 2016), as well as the effector empty vector, were unable to activate any of the reporter genes.

### Overexpression of *EgMYB88* or *EgMYB88-EAR* does not show effects in *Arabidopsis*

**Figure 6:**
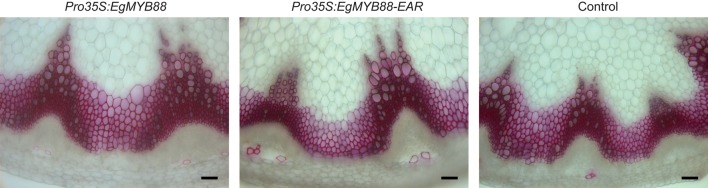
**Transverse sections of inflorescence stems of transgenic ***Arabidopsis*** plants overexpressing ***EgMYB88*** either as a native form or fused to an EAR motif**. Detail of *Arabidopsis* inflorescence stems where lignin are stained in red by phloroglucinol-HCl. Scale bars: 50 μm. Images shown are representative of the three independent lines analyzed for each construction (*Pro35S:EgMYB88* and *Pro35S:EgMYB88-EAR*, specified in Figure S1).

As a first step to functionally characterize *EgMYB88 in planta*, we used the 35S Cauliflower Mosaic Virus promoter promoter (*Pro35S*) to overexpress in *Arabidopsis* either its native form (*Pro35S:EgMYB88*) or a dominant repression form (*Pro35S:EgMYB88-EAR*). The fusion with the EAR motif was previously shown to efficiently convert transcriptional activators into strong repressors (Hiratsu et al., 2003). Several independent transgenic plants were obtained for each of the two constructs, which were phenotypically similar to the controls (wild-type plants and lines containing empty vectors) after visual inspection. We selected three lines for each construct (Figure S5) in order to further characterize their phenotypes by performing histochemical and biochemical analyses. Sections at the basis of the inflorescence stems were stained in red with phloroglucinol-HCl, a common stain for lignin. No differences in the staining intensity nor in the xylem structure were observed in the *Pro35S:EgMYB88* and/or *Pro35S:EgMYB88-EAR* lines compared to controls (Figure 6). Lignin contents assessed using the acetyl bromide method on extractive-free cell walls of *Arabidopsis* inflorescence stems were similar in *Pro35S:EgMYB88, Pro35S:EgMYB88-EAR* lines and controls (Table 1), in agreement with the histological observations. In order to have a wider overview of cell wall composition, we performed Py-GC/MS analysis of non-extracted *Arabidopsis* hypocotyls. The comparison between the transgenic lines and the controls did not reveal any differences (Table S3).

**Table 1:** **Lignin content in ***Arabidopsis*** inflorescence stems evaluated by acetyl bromide**.

	***Pro35S:EgMYB88***	***Pro35S:EgMYB88-EAR***	**Controls**
Acetyl bromide lignin (%)	15.48 ± 1.55	14.66 ± 0.45	15.37 ± 0.59

*Data represent the means ± standard deviation of three biological replicates for Pro35S:EgMYB88 and Pro35S:EgMYB88-EAR and five biological replicates for controls. Each biological replicate consisted of a pool of five plants from the same line (selected lines are specified in Figure S1). Acetyl bromide lignin is expressed as % of dry weight. No statistical differences were found using a Student's t test with a P-value < 0.05*.

### Lignin structure is altered in poplars overexpressing *EgMYB88* while lignin content is reduced when overexpressing *EgMYB88-EAR*

**Figure 7:**
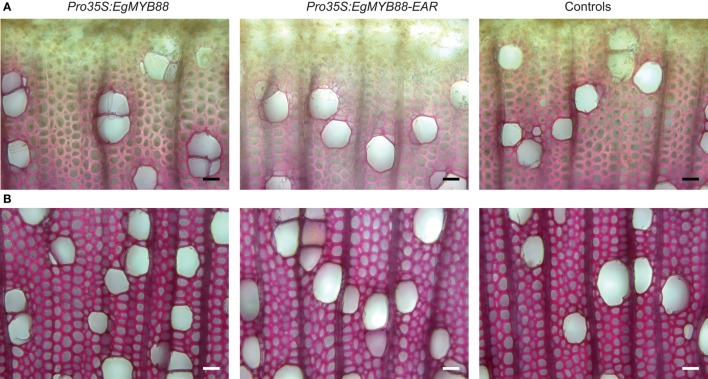
**Transverse sections of stems from transgenic poplar plants overexpressing ***EgMYB88*** either as a native form or fused to an EAR motif**. **(A)** Detail of the cambial region and of the first layers of differentiating xylem cells. **(B)** Detail of mature xylem cells. Sections were stained using phloroglucinol-HCl which stains the lignin in red. Scale bars: 25 μm. Images shown are representative of the three independent lines analyzed for each construction (*Pro35S:EgMYB88* and *Pro35S:EgMYB88-EAR*, specified in Figure S2). One single control representative of the two poplar batches is shown.

Poplar plants were transformed with the same kind of constructs, i.e., *Pro35S:EgMYB88* and *Pro35S:EgMYB88-EAR*. Transgenic lines generated *in vitro* did not show any visible differences as compared to control lines. Three independent lines per construct (Figure S6) were acclimated and transferred into the greenhouse for a deeper characterization by histochemical and biochemical analyses. The *Pro35S:EgMYB88* and *Pro35S:EgMYB88-EAR* plants were grown in two separate batches together with their appropriate controls. None of the lines showed differences in their overall xylem structure, including cell wall thickness (observed in mature xylem cells as well as in differentiating xylem close to the vascular cambium), vessel frequency and size (Figure 7). The intensity of the phloroglucinol-HCl staining of *Pro35S:EgMYB88* xylem was similar to that of their controls, whereas a weaker red color was observed in the xylem of *Pro35S:EgMYB88-EAR* lines, suggesting a reduction of the lignin content (Figure 7).

These histochemical observations were confirmed by Klason lignin quantification (Table 2). The insoluble lignin content of *Pro35S:EgMYB88* poplar plants was unchanged as compared to their corresponding controls (Table 2) while the *Pro35S:EgMYB88-EAR* poplar plants exhibited a small but significant reduction (6%) of the Klason lignin content (Table 3). Lignin structure was then analyzed by thioacidolysis, which specifically targets the lignin units (S and G) involved only in labile β-O-4 linkages (Méchin et al., 2014). When normalized relative to the Klason lignin content, the thioacidolysis yield was not significantly different between the *Pro35S:EgMYB88* lines and their controls, meaning that the total amount of G and S lignin units involved only in β-O-4 linkages was unaffected (Table 2). However, *Pro35S:EgMYB88* plants showed a significant different proportion of lignin units, with an increase in S units and a decrease in G units, resulting in a higher S/G ratio (13% higher than controls, Table 2). The thioacidolysis yield was not affected in the *Pro35S:EgMYB88-EAR* poplar xylem, but the proportion of S and G units were slightly affected resulting in a tendency of lower S/G ratio, although not statistically significant (Table 3).

**Table 2:** **Lignin content and structure in stems of ***Pro35S:EgMYB88*** poplar transgenic lines evaluated by Klason (KL) and thioacidolysis**.

**Lines**	**% KL**	**Lignin derived S and G thioacidolysis monomers: total yield and relative molar frequencies**			
		**Total yield (μmol/g KL)**	**% S**	**% G**	**S/G**
*Pro35S:EgMYB88*	17.49 ± 0.59	2890 ± 357	**64.6** ± 1.9^*^	**35.4** ± 1.9^*^	**1.83** ± 0.16^*^
Controls	17.96 ± 0.91	2949 ± 1042	61.6 ± 3.1	38.5 ± 3.1	1.62 ± 0.21

*Data represent the means ± standard deviation of eight Pro35S:EgMYB88 poplar (i.e., two-three individual plants for each of the three selected lines specified in Figure S2A) and seven control plants. Klason lignin is expressed as % of dry weight, and thioacidolysis yield is expressed relative to Klason lignin. Statistics were calculated with Student's t-test*,

* *P-value < 0.05*.

*Significant differences relative to the control values are highlighted in bold*.

**Table 3:** **Lignin content and structure in stems of ***Pro35S:EgMYB88-EAR*** poplar transgenic lines evaluated by Klason (KL) and thioacidolysis**.

**Lines**	**% KL**	**Lignin derived S and G thioacidolysis monomers: total yield and relative molar frequencies**			
		**Total yield (μmol / g KL)**	**% S**	**% G**	**S/G**
*Pro35S:EgMYB88-EAR*	**18.26** ± 1.27^*^	2746 ± 726	59.9 ± 5.1	40.1 ± 5.1	1.53 ± 0.35
Controls	19.48 ± 1.01	3135 ± 455	61.6 ± 1.9	38.5 ± 1.9	1.61 ± 0.13

*Data represent the means ± standard deviation of nine Pro35S:EgMYB88-EAR poplar (i.e., three individual plants for each of the three selected lines specified in Figure S2B) and nine control plants for Klason lignin, whereas seven Pro35S:EgMYB88-EAR poplar (i.e., two-three individual plants for each of the three selected lines specified in Figure S2B) and nine control plants were used for thioacidolysis. Klason lignin is expressed as % of dry weight, and thioacidolysis yield is expressed relative to Klason lignin. Statistics were calculated with Student's t-test*,

* *P-value < 0.05*.

*Significant differences relative to the control values are highlighted in bold*.

### Overexpression of *EgMYB88* and *EgMYB88-EAR* have distinct but not strictly opposite effects on poplar secondary metabolism

We also analyzed the effects of *EgMYB88* and *EgMYB88-EAR* on the profiles of soluble secondary metabolites in debarked stems of transgenic poplar lines. The Liquid Chromatography with photoDiode Array Detection–Mass Spectrometry (LC-DAD-MS) analysis of the water-methanol extracts revealed a complex composition of molecules absorbing at 280 nm. Among them, the 21 main different peaks were quantified, of which 14 could be attributed to a compound name or family (Figure S7).

The total content of phenolic secondary metabolites was increased by 12% in *Pro35S:EgMYB88* plants compared to controls. This increase was mostly due to salicinoid phenolic glycosides and to the flavonoid catechin, although some unidentified compounds were also increased (Table 4, Table S4). The three salicinoid phenolic glycosides detected were tremulacin, salicortin, and salireposide, which increased by 87, 70, and 56%, respectively as compared to controls. The level of the flavonoid catechin was also strongly increased (about 2.6 fold compared to controls). In the *Pro35S:EgMYB88-EAR* poplar plants, the total content of secondary metabolites was not significantly different from controls, neither were the contents of phenolic glycosides (Table 5, Table S5). However, the contents of catechin and oligolignols were significantly reduced by about 40% and 18%, respectively. The oligolignols G(8-O-4)SP(8-5)G [coniferyl alcohol (8-O-4) sinapyl *p*-hydroxybenzoate (8-5) coniferyl alcohol], G(8-O-4)SP(8-5)G′ [coniferyl alcohol (8-O-4) sinapyl *p*-hydroxybenzoate (8-5) coniferaldehyde] and a putative oligolignol not yet identified, were reduced by 35, 36, and 24%, respectively.

**Table 4:** **Metabolic profiling in stems of ***Pro35S:EgMYB88*** transgenic poplar lines**.

**Compounds name**	***Pro35S:EgMYB88***		**Controls**
	**Sum peak area (x1000)**	**Ratio**	**Sum peak area (x1000)**
Total metabolite signal	**4175.7** ± 52.4^***^	**1.1**	3721.7 ± 122.2
Catechin (flavonoid)	**80.3** ± 26.8^*^	**2.6**	30.4 ± 3.1
Sinapaldehyde	337.0 ± 65.6	1.2	276.4 ± 16.0
Salicinoid phenolic glycosides	**242.4** ± 26.4^**^	**1.7**	142.9 ± 3.7
Salicortin	**91.7** ± 13.7^**^	**1.7**	53.8 ± 1.9
Salireposide	**80.3** ± 4.6^***^	**1.6**	51.6 ± 0.7
Tremulacin	**70.4** ± 11.4^**^	**1.9**	37.6 ± 2.8
Oligolignols	498.1 ± 76.8	1.0	515.3 ± 30.1
G(8-O-4)S(8-5)G	102.4 ± 12.5	0.9	110.3 ± 7.5
G(8-O-4)S(8-5)G′	102.5 ± 9.4	0.9	108.7 ± 8.7
G(8-O-4)G(8-O-4)S(8-8)S	39.1 ± 7.4	1.0	39.7 ± 7.5
G(8-O-4)SP(8-5)G	93.8 ± 28.1	1.2	78.5 ± 17.3
G(8-O-4)SP(8-5)G′	36.2 ± 6.1	1.0	36.7 ± 10.6
Putative oligolignol	124.1 ± 36.8	0.9	133.5 ± 21.2
Unidentified compounds	**404.5** ± 12.2^***^	**1.3**	318.9 ± 9.0

*Data represent the means ± standard deviation of three Pro35S:EgMYB88 poplar (each Pro35S:EgMYB88 plant belongs to one of the three selected independent lines specified in Figure S2A) and three control plants. Statistics were calculated with Student's t-test*,

*** *P-value < 0.001*,

** *P-value < 0.01*,

* *P < 0.05*.

*Significant differences relative to controls are highlighted in bold. G(8–O–4)S(8–5)G consists in coniferyl alcohol (8-O-4) sinapyl alcohol (8-5) coniferyl alcohol; G(8–O–4)S(8–5)G′ consists in coniferyl alcohol (8-O-4) sinapyl alcohol (8-5) coniferaldehyde; G(8–O–4)G(8–O–4)S(8–8)S consists in coniferyl alcohol (8-O-4) coniferyl alcohol (8-O-4) sinapyl alcohol (8-8) sinapyl alcohol; G(8–O–4)SP(8–5)G consists in coniferyl alcohol (8-O-4) sinapyl p-hydroxybenzoate (8-5) coniferyl alcohol; G(8–O–4)SP(8–5)G' consists in coniferyl alcohol (8-O-4) sinapyl p-hydroxybenzoate (8-5) coniferaldehyde*.

**Table 5:** **Metabolic profiling in stems of ***Pro35S:EgMYB88-EAR*** transgenic poplar lines**.

**Compounds name**	***Pro35S:EgMYB88-EAR***		**Controls**
	**Sum peak area (x1000)**	**Ratio**	**Sum peak area (x1000)**
Total metabolite signal	3704.9 ± 159.3	1.0	3806.9 ± 312.3
Catechin (flavonoid)	**14.2** ± 3.6^*^	**0.6**	24.5 ± 4.9
Sinapaldehyde	373.3 ± 60.2	1.1	342.9 ± 32.5
Salicinoid phenolic glycosides	159.9 ± 32.0	1.0	155.4.2 ± 45.0
Salicortin	52.7 ± 13.9	0.9	59.6 ± 16.6
Salireposide	65.4 ± 11.9	0.9	70.5 ± 19.2
Tremulacin	41.9 ± 10.0	0.9	47.1 ± 24.1
Oligolignols	**520.4** ± 15.2^**^	**0.8**	639.8 ± 28.2
G(8-O-4)S(8-5)G	108.6 ± 1.3	1.0	111.1 ± 13.7
G(8-O-4)S(8-5)G′	78.2 ± 3.2	1.1	72.6 ± 19.2
G(8-O-4)G(8-O-4)S(8-8)S	23.4 ± 1.8	1.0	22.6 ± 4.9
G(8-O-4)SP(8-5)G	**95.5** ± 10.7^*^	**0.7**	146.3 ± 21.6
G(8-O-4)SP(8-5)G′	**23.9** ± 2.5^**^	**0.6**	37.2 ± 3.4
Putative oligolignol	**190.8** ± 8.5^*^	**0.8**	250.2 ± 35.1
Unidentified compounds	365.4 ± 18.7	1.0	368.1 ± 33.0

*Data represent the means ± standard deviation of three Pro35S:EgMYB88-EAR poplar (each Pro35S:EgMYB88-EAR plant belongs to one of the three selected independent lines specified in Figure S2B) and four control plants. Statistics were calculated with Student's t-test*,

** *P-value < 0.01*,

* *P < 0.05*.

*Significant differences relative to controls are highlighted in bold. G(8–O–4)S(8–5)G consists in coniferyl alcohol (8-O-4) sinapyl alcohol (8-5) coniferyl alcohol; G(8–O–4)S(8–5)G' consists in coniferyl alcohol (8-O-4) sinapyl alcohol (8-5) coniferaldehyde; G(8–O–4)G(8–O–4)S(8–8)S consists in coniferyl alcohol (8-O-4) coniferyl alcohol (8-O-4) sinapyl alcohol (8-8) sinapyl alcohol; G(8–O–4)SP(8–5)G consists in coniferyl alcohol (8-O-4) sinapyl p-hydroxybenzoate (8-5) coniferyl alcohol; G(8–O–4)SP(8–5)G' consists in coniferyl alcohol (8-O-4) sinapyl p-hydroxybenzoate (8-5) coniferaldehyde*.

### The transcript levels of phenylpropanoid metabolism genes show opposite tendencies in *EgMYB88* and *EgMYB88-EAR* transgenic poplars

**Figure 8:**
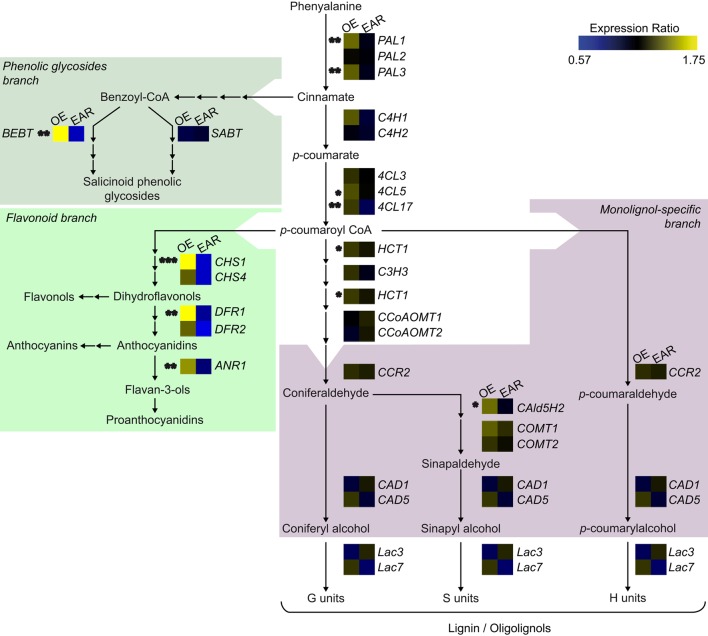
**Transcript abundance of genes involved in the phenylpropanoid metabolism in transgenic poplars overexpressing ***EgMYB88*** either as a native form or fused to an EAR motif**. The genes are placed in a schematic representation of the pathways leading to lignin, flavonoids, and salicinoid phenolic glycosides, where relevant intermediates are shown (adapted from Takos et al., 2006; Peng et al., 2008; Jaakola, 2013; Carocha et al., 2015; Chedgy et al., 2015; Wang et al., 2015). Transcript abundance was expressed as a ratio of the abundance of a given gene in transgenic poplar plants, either overexpressing *EgMYB88* (OE, shown at the left of the heatmap) or *EgMYB88-EAR* (EAR, shown at the right of the heat map), respective to its abundance in the corresponding controls. Values were calculated as the means of nine *Pro35S:EgMYB88* (i.e., three plants for each of the three selected independent transgenic lines, specified in Figure S2A) and nine respective control poplar plants, or eight *Pro35S:EgMYB88-EAR* (i.e., two-three plants for each of the three selected independent transgenic lines, specified in Figure S2B) and seven respective control poplar plants. Statistical significance was calculated with Student's *t*-test, ^***^P-value < 0.001, ^**^P-value < 0.01, ^*^P-value < 0.05. Gene names, expression data, and orthologs in other species are detailed in Tables 6, 7, Table S1.

To investigate the main modifications in gene expression induced by the overexpression of E*gMYB88* and *EgMYB88-EAR* in transgenic poplars, we analyzed the transcript levels of key genes involved in the phenylpropanoid metabolism and in cambium activity and patterning by microfluidic qPCR. In general, as can be seen in the overall picture of Figure 8, *Pro35S:EgMYB88* and *Pro35S:EgMYB88-EAR* showed opposed tendencies, meaning that genes induced in one transgenic type tend to be repressed in the other, and *vice versa*. However, whereas transcript abundance levels were clearly altered in *Pro35S:EgMYB88*, they were only moderately altered in *Pro35S:EgMYB88-EAR* plants (Tables 6, 7).

**Table 6:** **Transcript abundance of key genes in the stems of ***Pro35S:EgMYB88*** poplar plants**.

**Short name**	**Accession code**	**Full name**	**Transcript abundance**		
			***EgMYB88***	**Ratio**	**Control**
**PHENYLPROPANOID METABOLISM**					
*PAL1*	Potri.006G126800	*phenylalanine ammonia-lyase 1*	**1.34** ± 0.21^**^	**1.31**	1.02 ± 0.23
*PAL2*	Potri.008G038200	*phenylalanine ammonia-lyase 2*	1.04 ± 0.14	1.03	1.01 ± 0.18
*PAL3*	Potri.016G091100	*phenylalanine ammonia-lyase 3*	**1.31** ± 0.19^**^	**1.28**	1.02 ± 0.20
*C4H1*	Potri.013G157900	*cinnamate 4-hydroxylase 1*	1.31 ± 0.38	1.26	1.03 ± 0.26
*C4H2*	Potri.019G130700	*cinnamate 4-hydroxylase 2*	0.98 ± 0.18	0.97	1.00 ± 0.13
*4CL3*	Potri.001G036900	*4-Coumarate:CoA ligase 3*	1.18 ± 0.19	1.14	1.03 ± 0.27
*4CL5*	Potri.003G188500	*4-coumarate:CoA ligase 5*	**1.26** ± 0.20^*^	**1.23**	1.02 ± 0.24
*4CL17*	Potri.012G095000	*4-coumarate:CoA ligase 17*	**1.19** ± 0.08^**^	**1.18**	1.01 ± 0.14
*C3H3*	Potri.006G033300	*p-coumarate 3-hydroxylase 3*	1.19 ± 0.12	1.15	1.03 ± 0.28
*HCT1*	Potri.003G183900	*shikimate O-hydroxycinnamoyltransferase 1*	**1.18** ± 0.16^*^	**1.17**	1.01 ± 0.14
*CCoAOMT1*	Potri.009G099800	*caffeoyl CoA 3-O-methyltransferase 1*	1.02 ± 0.21	0.99	1.03 ± 0.24
*CCoAOMT2*	Potri.001G304800	*caffeoyl CoA 3-O-methyltransferase 2*	0.97 ± 0.34	0.93	1.04 ± 0.29
*CCR2*	Potri.003G181400	*cinnamoyl CoA reductase 2*	1.16 ± 0.23	1.14	1.02 ± 0.19
*COMT1*	Potri.015G003100	*caffeate/5-hydroxyferulate O-methyltransferase 1*	1.30 ± 0.45	1.28	1.02 ± 0.17
*COMT2*	Potri.012G006400	*caffeate/5-hydroxyferulate O-methyltransferase 2*	1.18 ± 0.21	1.15	1.02 ± 0.22
*CAld5H2*	Potri.007G016400	*coniferaldehyde 5-hydroxylase*	**1.34** ± 0.28^*^	**1.32**	1.01 ± 0.21
*CAD1*	Potri.009G095800	*Cinnamyl alcohol dehydrogenase 1*	0.93 ± 0.34	0.90	1.03 ± 0.24
*CAD5*	Potri.009G062800	*Cinnamyl alcohol dehydrogenase 5*	1.17 ± 0.32	1.16	1.01 ± 0.18
*CSE1*	Potri.003G059200	*caffeoyl shikimate esterase 1*	1.05 ± 0.19	1.04	1.01 ± 0.09
*CSE2*	Potri.001G175000	*caffeoyl shikimate esterase 2*	1.01 ± 0.13	1.00	1.01 ± 0.13
*lac3*	Potri.010G193100	*laccase 3*	0.86 ± 0.14	0.85	1.01 ± 0.17
*lac17*	Potri.001G401300	*laccase 17*	1.18 ± 0.31	1.16	1.02 ± 0.18
*CHS1*	Potri.014G145100	*chalcone synthase 1*	**2.08** ± 0.62^***^	**2.01**	1.04 ± 0.29
*CHS4*	Potri.003G176700	*chalcone synthase 4*	1.32 ± 0.37	1.28	1.03 ± 0.25
*DFR1*	Potri.002G033600	*dihydroflavonol-4-reductase 1*	**1.79** ± 0.63^**^	**1.75**	1.02 ± 0.22
*DFR2*	Potri.005G229500	*dihydroflavonol-4-reductase 2*	1.38 ± 0.35	1.29	1.07 ± 0.40
*ANR1*	Potri.004G030700	*anthocyanidin reductase 1*	**1.46** ± 0.37^**^	**1.44**	1.02 ± 0.19
*BEBT*	Potri.019G043600	*benzoyl-CoA:benzyl alcohol O-benzoyltransferase*	**2.77** ± 1.22^**^	**2.35**	1.18 ± 0.75
*SABT*	Potri.013G074500	*benzoyl-CoA:salicyl alcohol O-benzoyltransferase*	0.94 ± 0.47	0.88	1.06 ± 0.34
**CAMBIUM ACTIVITY AND PATTERNING**					
*HB2*	Potri.004G211300	*HD-Zip III family protein HB2/PopREV*	**0.78** ± 0.17^**^	**0.78**	1.00 ± 0.16
*HB4*	Potri.001G372300	*HD-Zip III family protein HB4*	**0.79** ± 0.15^*^	**0.78**	1.02 ± 0.22
*HB7*	Potri.018G045100	*HD-Zip III family protein HB7*	1.00 ± 0.09	0.99	1.01 ± 0.15
*HB8*	Potri.006G237500	*HD-Zip III family protein HB8*	1.07 ± 0.15	1.06	1.01 ± 0.14
*PIN1b*	Potri.015G038700	*PIN-FORMED 1b*	**0.69** ± 0.18^**^	**0.67**	1.03 ± 0.23
*PIN1d*	Potri.016G035300	*PIN-FORMED 1d*	1.07 ± 0.36	1.00	1.07 ± 0.37
*WOX4a*	Potri.002G124100	*WUSCHEL-related homeobox family protein 4a*	0.84 ± 0.51	0.80	1.04 ± 0.29
*WOX4b*	Potri.014G025300	*WUSCHEL-related homeobox family protein 4b*	0.79 ± 0.43	0.75	1.06 ± 0.34
*PXY*	Potri.003G107600	*PHLOEM INTERCALATED WITH XYLEM*	1.29 ± 0.34	1.23	1.04 ± 0.33
*CLE41*	Potri.012G019400	*CLV3/ESR1-LIKE 41*	1.19 ± 1.44	0.73	1.64 ± 2.54
***EgMYB88*** **POPLAR ORTHOLOGS**					
*MYB011*	Potri.001G169600	*R2R3-MYB transcription factor 11*	1.29 ± 0.30	1.24	1.04 ± 0.31
*MYB171*	Potri.003G064600	*R2R3-MYB transcription factor 171*	**0.68** ± 0.17^**^	**0.65**	1.05 ± 0.33

*Short name, full name and accession number (P. trichocarpa genome v3.0) are indicated for each gene. Transcript abundance (mean ± standard deviation) was calculated from nine Pro35S:EgMYB88 poplar (i.e., three plants for each of the three selected independent transgenic lines specified in Figure S2A) and nine control plants. Ratios of transcript abundance in transgenic poplars relative to controls are also indicated. Statistics were calculated with Student's t-test*,

*** *P-value < 0.001*,

** *P-value < 0.01*,

* *P-value < 0.05*.

*Significant differences relative to the control values are highlighted in bold. Primer sequences are indicated in Table S1*.

**Table 7:** **Transcript abundance of key genes in the stems of ***Pro35S:EgMYB88-EAR*** poplar plants**.

**Short name**	**Accession code**	**Full name**	**Transcript abundance**		
			***EgMYB88-EAR***	**Ratio**	**Controls**
**PHENYLPROPANOID METABOLISM**					
*PAL1*	Potri.006G126800	*phenylalanine ammonia-lyase 1*	0.98 ± 0.22	0.96	1.02 ± 0.19
*PAL2*	Potri.008G038200	*phenylalanine ammonia-lyase 2*	1.02 ± 0.23	0.99	1.04 ± 0.42
*PAL3*	Potri.016G091100	*phenylalanine ammonia-lyase 3*	0.98 ± 0.22	0.95	1.03 ± 0.22
*C4H1*	Potri.013G157900	*cinnamate 4-hydroxylase 1*	0.93 ± 0.34	0.90	1.02 ± 0.28
*C4H2*	Potri.019G130700	*cinnamate 4-hydroxylase 2*	0.94 ± 0.13	0.93	1.01 ± 0.24
*4CL3*	Potri.001G036900	*4-Coumarate:CoA ligase 3*	1.01 ± 0.13	1.00	1.01 ± 0.19
*4CL5*	Potri.003G188500	*4-coumarate:CoA ligase 5*	1.02 ± 0.14	1.01	1.01 ± 0.12
*4CL17*	Potri.012G095000	*4-coumarate:CoA ligase 17*	0.88 ± 0.09	0.86	1.02 ± 0.21
*C3H3*	Potri.006G033300	*p-coumarate 3-hydroxylase 3*	0.96 ± 0.15	0.96	1.00 ± 0.15
*HCT1*	Potri.003G183900	*shikimate O-hydroxycinnamoyltransferase 1*	1.08 ± 0.18	1.06	1.01 ± 0.24
*CCoAOMT1*	Potri.009G099800	*caffeoyl CoA 3-O-methyltransferase 1*	1.13 ± 0.25	1.10	1.02 ± 0.32
*CCoAOMT2*	Potri.001G304800	*caffeoyl CoA 3-O-methyltransferase 2*	1.22 ± 0.21	1.06	1.15 ± 0.53
*CCR2*	Potri.003G181400	*cinnamoyl CoA reductase 2*	1.10 ± 0.22	1.09	1.01 ± 0.12
*COMT1*	Potri.015G003100	*caffeate/5-hydroxyferulate O-methyltransferase 1*	1,15 ± 0.30	1.13	1.01 ± 0.16
*COMT2*	Potri.012G006400	*caffeate/5-hydroxyferulate O-methyltransferase 2*	1.06 ± 0.20	1.04	1.02 ± 0.25
*CAld5H2*	Potri.007G016400	*coniferaldehyde 5-hydroxylase*	0.96 ± 0.14	0.95	1.01 ± 0.11
*CAD1*	Potri.009G095800	*Cinnamyl alcohol dehydrogenase 1*	1.32 ± 0.29	1.07	1.23 ± 0.65
*CAD5*	Potri.009G062800	*Cinnamyl alcohol dehydrogenase 5*	0.95 ± 0.26	0.91	1.05 ± 0.26
*CSE1*	Potri.003G059200	*caffeoyl shikimate esterase 1*	0.99 ± 0.22	0.93	1.06 ± 0.38
*CSE2*	Potri.001G175000	*caffeoyl shikimate esterase 2*	1.01 ± 0.17	1.00	1.01 ± 0.13
*lac3*	Potri.010G193100	*laccase 3*	1.12 ± 0.16	1.11	1.01 ± 0.14
*lac17*	Potri.001G401300	*laccase 17*	0.88 ± 0.29	0.83	1.07 ± 0.40
*CHS1*	Potri.014G145100	*chalcone synthase 1*	0.78 ± 0.34	0.66	1.18 ± 0.80
*CHS4*	Potri.003G176700	*chalcone synthase 4*	0.95 ± 0.48	0.67	1.42 ± 1.46
*DFR1*	Potri.002G033600	*dihydroflavonol-4-reductase 1*	0.88 ± 0.42	0.75	1.17 ± 0.83
*DFR2*	Potri.005G229500	*dihydroflavonol-4-reductase 2*	0.81 ± 0.38	0.62	1.32 ± 1.16
*ANR1*	Potri.004G030700	*anthocyanidin reductase 1*	0.85 ± 0.23	0.81	1.06 ± 0.36
*BEBT*	Potri.019G043600	*benzoyl-CoA:benzyl alcohol O-benzoyltransferase*	0.85 ± 0.54	0.68	1.25 ± 0.92
*SABT*	Potri.013G074500	*benzoyl-CoA:salicyl alcohol O-benzoyltransferase*	1.22 ± 0.79	0.91	1.33 ± 1.26
**CAMBIUM ACTIVITY AND PATTERNING**					
*HB2*	Potri.004G211300	*HD-Zip III family protein HB2/PopREV*	1.32 ± 0.29	1.24	1.07 ± 0.39
*HB4*	Potri.001G372300	*HD-Zip III family protein HB4*	1.27 ± 0.33	1.16	1.09 ± 0.48
*HB7*	Potri.018G045100	*HD-Zip III family protein HB7*	1.12 ± 0.15	1.11	1.02 ± 0.19
*HB8*	Potri.006G237500	*HD-Zip III family protein HB8*	1.11 ± 0.11	1.09	1.03 ± 0.25
*PIN1b*	Potri.015G038700	*PIN-FORMED 1b*	1.12 ± 0.32	1.07	1.05 ± 0.37
*PIN1d*	Potri.016G035300	*PIN-FORMED 1d*	1.05 ± 0.17	1.02	1.03 ± 0.19
*WOX4a*	Potri.002G124100	*WUSCHEL-related homeobox family protein 4a*	1.42 ± 0.55	1.29	1.10 ± 0.48
*WOX4b*	Potri.014G025300	*WUSCHEL-related homeobox family protein 4b*	1.57 ± 0.63	1.33	1.19 ± 0.72
*PXY*	Potri.003G107600	*PHLOEM INTERCALATED WITH XYLEM*	1.21 ± 0.44	1.17	1.04 ± 0.33
*CLE41*	Potri.012G019400	*CLV3/ESR1-LIKE 41*	1.05 ± 0.23	0.91	1.15 ± 0.65
***EgMYB88*** **POPLAR ORTHOLOGS**					
*MYB011*	Potri.001G169600	*R2R3-MYB transcription factor 11*	**1**.**52** ± **0**.58^*^	**1**.**49**	1.02 ± 0.15
*MYB171*	Potri.003G064600	*R2R3-MYB transcription factor 171*	1.25 ± 0.21	1.20	1.04 ± 0.32

*Short name, full name and accession number (P. trichocarpa genome v3.0) are indicated for each gene. Transcript abundance (mean ± standard deviation) was calculated from eight Pro35S:EgMYB88-EAR poplar (i.e., two-three plants for each of the three selected independent transgenic lines specified in Figure S2B) and seven control plants. Ratios of transcript abundance in transgenic poplars relative to controls are also indicated. Statistics were calculated with Student's t-test*,

* *P-value < 0.05*.

*Significant differences relative to the control values are highlighted in bold. Primer sequences are indicated in Table S1*.

Most of the genes involved in the biosynthesis of phenylpropanoids, including those of lignin, flavonoids, and phenolic glycosides branches, were induced either significantly or at least showed a tendency to be up-regulated in *Pro35S:EgMYB88* poplar plants (Figure 8, Table 6). In contrast, they were repressed in *Pro35S:EgMYB88-EAR* poplars although not significantly because of the high variation found among plants (Figure 8, Table 7). Interestingly, genes involved in the biosynthesis of flavonoids and salicinoid phenolic glycosides were the most induced ones in *Pro35S:EgMYB88* poplars (Figure 8, Table 6). The gene encoding benzoyl-CoA:benzyl alcohol *O*-benzoyltransferase (BEBT), an enzyme involved in the biosynthesis of salicortin and other salicinoid phenolic glycosides (Babst et al., 2010; Chedgy et al., 2015) was the most induced (2.4 fold induction), in agreement with the strong accumulation of phenolic glycosides found in these lines. The genes encoding chalcone synthase (CHS), dihydroflavonol-4-reductase (DFR), and anthocyanidin reductase (ANR), involved in the biosynthesis of flavonoids, were also strongly induced (Figure 8, Table 6), in agreement with the increase in catechin levels. The genes from the general phenylpropanoid pathway (phenylalanine ammonia-lyase, *PAL* and 4-coumarate:CoA ligase, *4CL*) were moderately induced in *Pro35S:EgMYB88* poplars as was ferulate 5-hydroxylase (*CAld5H*), involved in the biosynthesis of sinapyl alcohol which generate S lignin units, in agreement with the increase in the S/G ratio.

Most of the genes regulating the cambial activity tested in this study were repressed or showed a tendency to be repressed in *Pro35S:EgMYB88* poplars (Table 6), whereas they seemed to be induced in *Pro35S:EgMYB88-EAR* lines (Table 7). *HB2/PopREV* and *HB4*, respectively orthologs of *Arabidopsis REVOLUTA* and *PHABULOSA/PHAVOLUTA* (Zhu et al., 2013), and *PIN1b*, ortholog of *Arabidopsis PIN1* (Liu et al., 2014b), were significantly repressed in *EgMYB88* overexpressing poplars (22, 22, and 23% of repression, respectively). These genes are related to cambium activity and to the differentiation of vascular tissues in *Arabidopsis* (Gälweiler et al., 1998; Zhong and Ye, 1999) and poplar (Robischon et al., 2011).

Finally, we analyzed the transcript levels of the two poplar orthologs of *EgMYB88: MYB11* and *MYB171*. Unexpectedly, the two genes exhibited opposite behaviors depending on the transgenic lines type. For example, in *Pro35S:EgMYB88* plants, *MYB171* was clearly repressed (35% repression), whereas *MYB11* was not significantly affected (Table 6). In contrast, *MYB171* was not significantly altered in *Pro35S:EgMYB88-EAR* plants whereas *MYB11* was clearly induced (40% induction) (Table 7).

## Discussion

Our study focused on the R2R3 MYB genes from subgroup WPS-I which are present preferentially in woody perennial plants exhibiting secondary growth (Soler et al., 2015). To the best of our knowledge, none of the genes belonging to this subgroup has been functionally characterized so far in any species. In *Eucalyptus*, the six genes of this subgroup have not been tandem-duplicated in contrast to most of the genes belonging to the woody-preferential and woody-expanded subgroups (Soler et al., 2015). Five of the six WPS-I *Eucalyptus* genes and the four poplar genes are targets of miR828, the same miR which targets R2R3 MYBs from several subgroups, many being involved in the regulation of the anthocyanin and the lignin pathways (Xia et al., 2012). Moreover, miR828 was also shown to target *GhMYB2*, which is involved in cotton fiber development (Guan et al., 2014). In *Eucalyptus*, miR828 belong to a cluster of miRs regulated during reaction wood formation (Carocha and Paiva, personal communication) suggesting that WPS-I genes can be controlled post-transcriptionally in response to a mechanical stress. In addition, the transcriptional activities of the WPS-I genes could be modulated through interactions with other proteins, as suggested by the presence of a well conserved bHLH interacting motif among all the genes from the subgroup. Indeed, many R2R3-MYBs interact with bHLH and WD40 proteins, thereby specifying their functions (Ramsay and Glover, 2005). Such interactions were shown to contribute to the tight regulation of the flavonoid biosynthetic genes (Xu et al., 2015).

The transcript profiles of all *Eucalyptus MYBs* from WPS-I revealed a high expression in the cambial region, reinforcing the idea that they may be involved in some aspects of wood formation. We selected *EgrMYB88*, which had the highest expression and was also preferentially expressed in this tissue. We showed that EgMYB88 acts as an autoactivator in yeast, suggesting that it is also a transcriptional activator in plants. We functionally characterized *EgMYB88* in *Arabidopsis* and poplar, two model plants previously shown to be useful to decipher the function of *Eucalyptus MYB* genes (Legay et al., 2010). When overexpressed in *Arabidopsis*, either in its native form or fused to an EAR motif, *EgMYB88* did not produce any phenotype evaluated either at the macroscopic, histologic or biochemical levels. This lack of phenotype could be expected since this gene is absent from the Brassicaceae, suggesting that *EgMYB88* is not active in the herbaceous model A*rabidopsis* due to the lack of either appropriate DNA-binding elements in target genes and/or absence of protein partners necessary for the proper function of the gene. It is also possible that miR828 modulates *EgMYB88* expression in the transgenic plants resulting in moderate phenotypes in poplar and no phenotypes in *Arabidopsis*.

In poplar plants, EgMYB88 is able to directly or indirectly activate the phenylpropanoid pathway, as shown by the accumulation of catechin and salicinoid phenolic glycosides in the wood-forming tissues of *EgMYB88*-overexpressing plants, and also by the induction of many phenylpropanoid biosynthetic genes. The regulation of the phenylpropanoid pathway is also supported by the phenotype of poplars overexpressing *EgMYB88-EAR*, showing a decrease of catechin levels and lowered transcript levels of many phenylpropanoid biosynthetic genes. However, the phenotype of poplars overexpressing *EgMYB88-EAR* is not exactly the opposite of that of poplars overexpressing *EgMYB88*, as the former did not show changes in the levels of salicinoid phenolic glycosides, but a reduction in the level of soluble oligolignols concomitant with a reduction of lignin level. Oligolignols are soluble oligomers constituted of monolignols that polymerize similarly to lignin (Morreel et al., 2010; Vanholme et al., 2010) and, in general, plants that produce less lignin show a concomitant reduction of their content in oligolignols (Morreel et al., 2004; Damiani et al., 2005). Interestingly, the oligolignols that were repressed in *EgMYB88-EAR* overexpressing poplars were those containing sinapyl *p*-hydroxybenzoate (SP) compounds, formed by the acylation of a sinapyl alcohol with a *p*-hydroxybenzoic acid prior to their incorporation to the growing lignin polymer. SP compounds are present in lignin from poplar as well as other plants (Morreel et al., 2004). The fact that *EgMYB88-EAR* overexpressing poplars contain a significant reduction of oligolignols deriving from benzoic acid, whereas *EgMYB88* overexpressing plants show an important accumulation of salicinoid phenolic glycosides also deriving from benzoic acid suggests an unexpected link between these two phenylpropanoid branch pathways possibly mediated by *EgMYB88*.

Salicortin, tremulacin and salireposide are three salicinoid phenolic glycosides abundant in leaves and bark of Salicaceae, where they play a role as anti-herbivore defenses (Boeckler et al., 2011). They were shown to accumulate in the xylem ray cells of transgenic poplar silenced for *laccase3*, which was interpreted as a consequence of a defect in the polymerization of some phenolic compounds in the cell walls of neighboring xylem fibers without altering lignin levels (Ranocha et al., 2002). It is worth noting that recent studies have shown the incorporation of non-monolignol units in the lignin polymer. It is the case for instance of the flavonoid tricin in monocots (Lan et al., 2015). In poplar plants overexpressing *EgMYB88, laccase3* showed a tendency to be down-regulated in contrast to many of the phenylpropanoid genes tested which were in general induced. The lignin content of these plants was not affected but lignin structure was altered with an increased S/G ratio.

The biosynthesis of the salicinoids is poorly understood but their phenylpropanoid origin has been confirmed (Tsai et al., 2011). Two BADH-type acyltransferases have recently been proposed to be involved in the biosynthesis of the salicinoids: The benzoyl-CoA:salicyl alcohol *O*-benzoyltransferase (SABT) and the benzoyl-CoA:benzyl alcohol *O*-benzoyltransferase (BEBT) (Chedgy et al., 2015). The transcript encoding BEBT was the most induced in the xylem of poplar overexpressing *EgMYB88*, while that encoding SABT was not affected. This result strongly supports the hypothesis that benzyl benzoate could be the preferred intermediate in the synthesis of salicortin, tremulacin, and salireposide in poplar as proposed by Chedgy et al. (2015). Since salicinoid phenolic glycosides are taxonomically limited to the Salicaceae, *EgMYB88* is likely regulating the biosynthesis of other benzoic-acid derivative compounds within the phenylpropanoid metabolism in the cambial region of *Eucalyptus*.

Poplar lines overexpressing *EgMYB88* showed a notable induction of genes encoding enzymes involved in the biosynthesis of flavonoids, *CHS1, DFR1*, and *ANR1*, in agreement with the high levels of catechin. Catechin is a flavonoid of the flavan-3-ol subfamily found in many vascular plants, and also widely found as part of proanthocyanidins. Flavonoids have many roles in plants, like conferring protection against UV-B and oxidative stress, protection against pathogens and herbivores, facilitating plant reproduction and fertility, improving the resistance to toxic metals (Falcone Ferreyra et al., 2012), and also interfering with the polar auxin transport (Peer and Murphy, 2007; Kuhn et al., 2011). Flavan-3-ols are considered as markers of undifferentiated callus-like cells and their accumulation have been correlated to a lack of cambium cell differentiation (reviewed by Treutter, 2010). Taking into account that polar auxin transport is thought to be necessary for cambial activity and vascular tissue formation (Schuetz et al., 2013), it is tempting to hypothesize that some R2R3-MYB genes like *EgMYB88* could regulate the biosynthesis of some flavonoids to modify auxin transport in cambium and thus locally modify cambium activity in response to environmental factors (Soler et al., 2015). Supporting this hypothesis, genes presumably involved in polar auxin transport necessary for the cambium proliferation and differentiation of vascular tissues like *PIN1b* (Liu et al., 2014b), or in the cambium initiation and patterning of secondary vascular tissues like *HB2/PopREV* (Robischon et al., 2011), are repressed in *EgMYB88* overexpressing poplars. It is also worth noting that *Eucalyptus* supplemented with flavonoids show an increase of the lignin S/G ratio (Lepikson-Neto et al., 2013), similarly found in *EgMYB88* overexpressing poplar lines containing higher levels of catechin.

The two poplar orthologs of *EgMYB88*, which to the best of our knowledge have not been yet functionally characterized, showed unexpected opposite behaviors in the transgenic poplar lines. The transcript of *MYB171* was clearly repressed when *EgMYB88* was overexpressed, whereas that of *MYB11* was induced when the dominant repressive version of EgMYB88 was overexpressed. It is possible that *MYB11* and *MYB171* have only partially overlapping targets in poplar, explaining why poplar plants overexpressing a dominant repression form of *EgMYB88* (potentially strong repressor), did not show the opposite phenotypes of the ones overexpressing the native form (activator). One could hypothesize that *MYB171* is involved in the regulation of the phenolic glycosides and flavonoids pathways, and its transcription could be regulated by a negative regulatory loop when the production of these compounds is too high or to counterbalance the induction of its target genes, as it happens in *EgMYB88* overexpressing poplars. On the other hand, when *EgMYB88-EAR* is acting as a repressor, the transcript of *MYB11* is induced possibly to counterbalance the repressive effects on its target genes. These compensatory mechanisms could explain why the transcripts levels of genes significantly induced in *Pro35S:EgMYB88* exhibited only a tendency to be repressed in *Pro35S:EgMYB88-EAR*.

The regulation of the biosynthesis of phenylpropanoid-derived secondary metabolites and their relationship with lignin has not been yet properly studied in tissues like cambium and differentiating xylem. *EgMYB88* is a R2R3-MYB gene belonging to WPS-I, a group of genes preferentially found in woody perennials exhibiting secondary growth (Soler et al., 2015). The highly preferential expression of *EgMYB88* in vascular cambium makes it an outstanding candidate to study the regulation of wood formation. *EgMYB88* behaves as a transcriptional activator in yeast, but when overexpressed in *Arabidopsis* plants, no phenotype was observed. In contrast, the analyses of the transgenic poplar plants overexpressing *EgMYB88* indicate that this gene regulates the phenylpropanoid metabolism by controlling, either directly or indirectly, the biosynthesis of lignin, but also of flavonoids and salicinoid phenolic glycosides, mostly known for their protective roles. As genes involved in cambium proliferation and differentiation of vascular tissues are also altered in these transgenic poplar plants, it seems plausible that *EgMYB88* contributes to adapt wood formation depending on environmental conditions. This is particularly important because woody plants need to face a myriad of complex stresses over their long lifespans. The characterization of this gene using a homologous transformation system, such as the *Eucalyptus* transgenic hairy roots recently set up in our lab (Plasencia et al., 2016), together with the identification of its direct target genes, will enable to more accurately specify its role in the cambial region.

## Author contributions

MS, AP, RL, and JG designed the research. MS, AP, JL, EC, AD, NL, FM, and RL performed research. MS, AP, JL, EP, RL, and JG analyzed data. MS, AP, RL, and JG wrote the article. All authors read, corrected, and approved the article.

## Funding

This work was supported by the *Centre National pour la Recherche Scientifique* (CNRS), the University Paul Sabatier Toulouse III (UPS), the French Laboratory of Excellence project “TULIP” (ANR-10-LABX-41; ANR-11-IDEX-0002-02), and the TreeForJoules project (ANR-2010-KBBE-007-01). MS was supported by the postdoctoral fellowship “Beatriu de Pinós” (2009 BP-A 00185) from the *Departament d'Universitats, Recerca i Societat de la Informació de la Generalitat de Catalunya* and by grants from TreeForJoules and Tulip. AP by a PhD grant from the *Ministère de l'Education Nationale, de l'Enseignement Supérieur et de la Recherche*. JL by a postdoctoral fellowship from the Sao Paulo Research Foundation (FAPESP, 2013/17846-0). EC by a postdoctoral fellowship from CNPQ-Brazil (202228/2015-0).

### Conflict of interest statement

The authors declare that the research was conducted in the absence of any commercial or financial relationships that could be construed as a potential conflict of interest.

## Abbreviations

4CL: 4-coumarate:CoA ligase
ACT2: *actin2*
Ade: Adenine
ANR: anthocyanidin reductase
AurA: Aureobasidin A
BD: Gal4 DNA binding domain
BEBT, benzoyl-CoA: benzyl alcohol O-benzoyltransferase
bHLH: basic Helix-Loop-Helix proteins
Cald5H: ferulate 5-hydroxylase
CDC2: cell division cycle2
CHS: chalcone synthase
DFR: dihydroflavonol-4-reductase
EAR: Ethylene-responsive element binding factor-associated Amphiphilic Repression motif
G units: guaiacyl lignin units
G(8-O-4)SP(8-5)G: coniferyl alcohol (8-O-4) sinapyl p-hydroxybenzoate (8-5) coniferyl alcohol
G(8-O-4)SP(8-5)G′: coniferyl alcohol (8-O-4) sinapyl p-hydroxybenzoate (8-5) coniferaldehyde
GC/MS: Gas Chromatography/Mass Spectrometry
H units: *p*-hydroxyphenyl lignin units
His: Histidine
HK1, HK3, and HK11: Housekeeping genes 1, 3 and 11
IBA: indole-3-butyric acid
KL: Klason lignin
LC-DAD-MS: Liquid Chromatography with photoDiode Array Detection-Mass Spectrometry
PAL: phenylalanine ammonia-lyase
Pro35S: 35S Cauliflower Mosaic Virus promoter
Py-GC/MS: Pyrolysis–Gas Chromatography/Mass Spectrometry
RT-qPCR: RetroTranscriptase-quantitative Polymerase Chain Reaction
S units: syringyl lignin units
SABT: benzoyl-CoA: salicyl alcohol O-benzoyltransferase
SD: synthetically defined medium used in the yeast autoactivation tests
SP: sinapyl p-hydroxybenzoate
Trp: Tryptophan
U-HPLC: Ultra-High Performance Liquid Chromatography
UBQ: Ubiquitin
WPS: Woody Preferential Subgroups
X-α-Gal: 5-bromo-4-chloro-3-indolyl alpha-D-galactopyranoside.
