# Supplementary material for: The Woody-Preferential Gene EgMYB88 Regulates the Biosynthesis of Phenylpropanoid-Derived Compounds in Wood
Source: Front Plant Sci. 2016 Sep 22;7:1422. doi: 10.3389/fpls.2016.01422 (PMC5032791; doi:10.3389/fpls.2016.01422)
Supplement: Supplementary file 2 [file Presentation1.pdf]

Supplementary material: Figures S1-S7, Tables S2-S5

The woody-preferential gene *EgMYB88* regulates the biosynthesis of phenylpropanoid-derived compounds in wood

Marçal Soler, Anna Plasencia, Jorge Lepikson-Neto, Eduardo Leal Oliveira Camargo, Annabelle Dupas, Nathalie Ladouce, Edouard Pesquet, Fabien Mounet, Romain Larbat, Jacqueline Grima-Pettenati*

* **Correspondence:** Jacqueline Grima Pettenati: grima@lrsv.ups-tlse.fr

Figure S1. EgMYB88 transgene expression levels in several lines of *EgMYB88* (A) and *EgMYB88-EAR* (B) transformed *Arabidopsis* plants.

Figure S2. EgMYB88 transgene expression levels in several lines of *EgMYB88* (A) and *EgMYB88-EAR* (B) transformed poplar plants.

Figure S3. Physical position of the six genes from WPS-I on the 11 *E. grandis* chromosomes.

Figure S4. RNAseq expression values from the different poplar genes from WPS-I, extracted from PopGenIE (Sjödin et al., 2009) as part of an expression atlas of *P. tremula* (Sundell et al., 2015).

Figure S5. Macroscopic images of transgenic *Arabidopsis* plants grown in short-day conditions for four weeks (A) and until sampling (B).

Figure S6. Macroscopic image of transgenic poplar plants just before sampling.

Figure S7. Chromatogram profiles of the water-methanol extracts of poplar *Pro35S:EgMYB88* (A) and *Pro35S:EgMYB88-EAR* (B) with their respective controls.

Table S2. Amino acid similarities between the *E. grandis* and the *P. trichocarpa* R2R3-MYB sequences belonging to WPS-I.

Table S3. Relative amounts of the different categories of phenolics compounds identified by Py-GC/MS in non-extracted *Arabidopsis* hypocotyls.

Table S4. Complete metabolic profiling in stems of *Pro35S:EgMYB88* transgenic poplar lines.

Table S5. Complete metabolic profiling in stems of *Pro35S:EgMYB88-EAR* transgenic poplar lines.

Figure S1

A

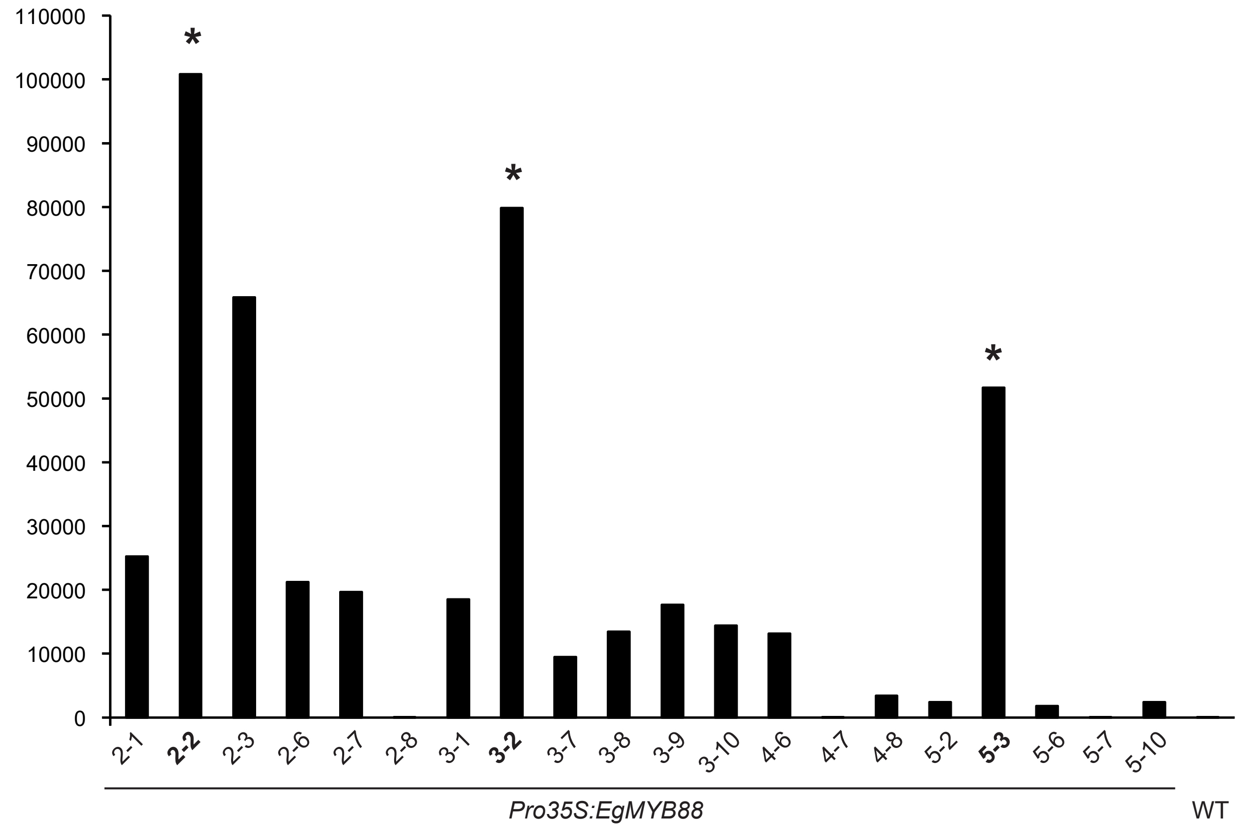

B

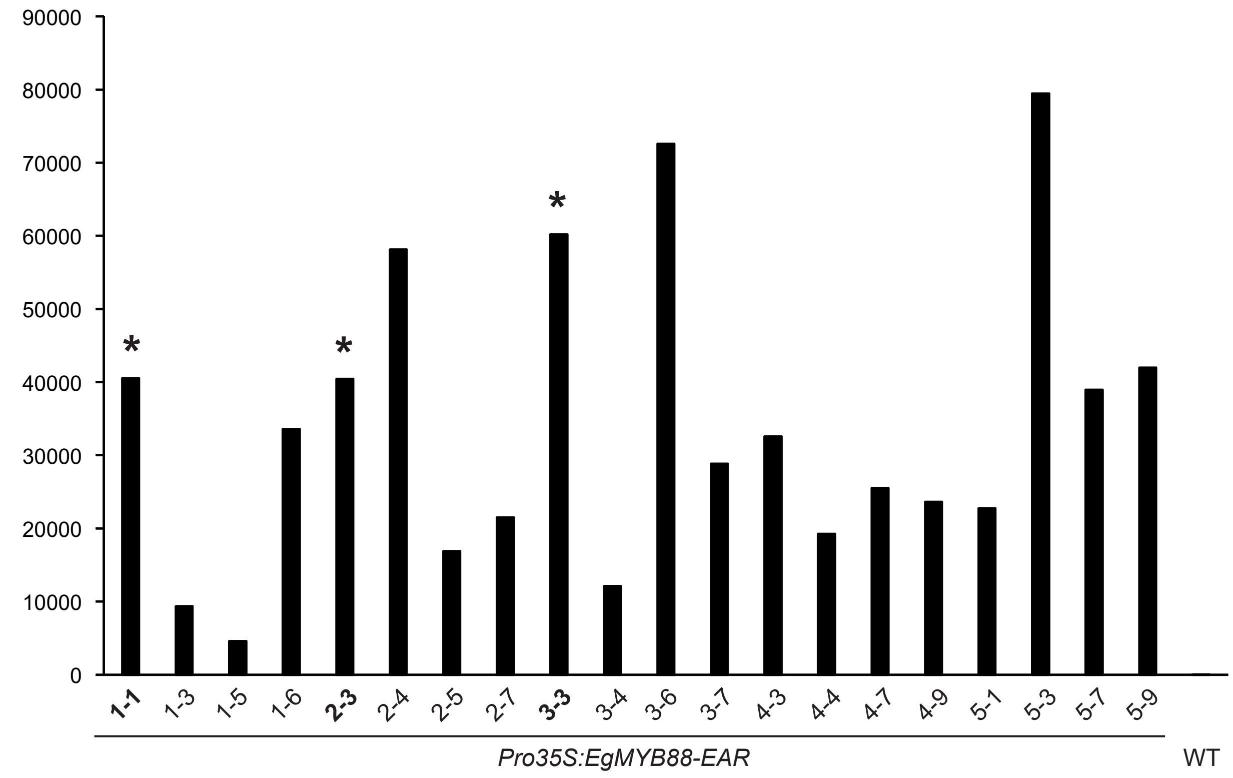

Figure S1. EgMYB88 transgene expression levels in several lines of *EgMYB88* (A) and *EgMYB88-EAR* (B) transformed *Arabidopsis* plants. Selected lines for detailed characterization of their phenotypes are highlighted in bold and with an asterisk.

Figure S2

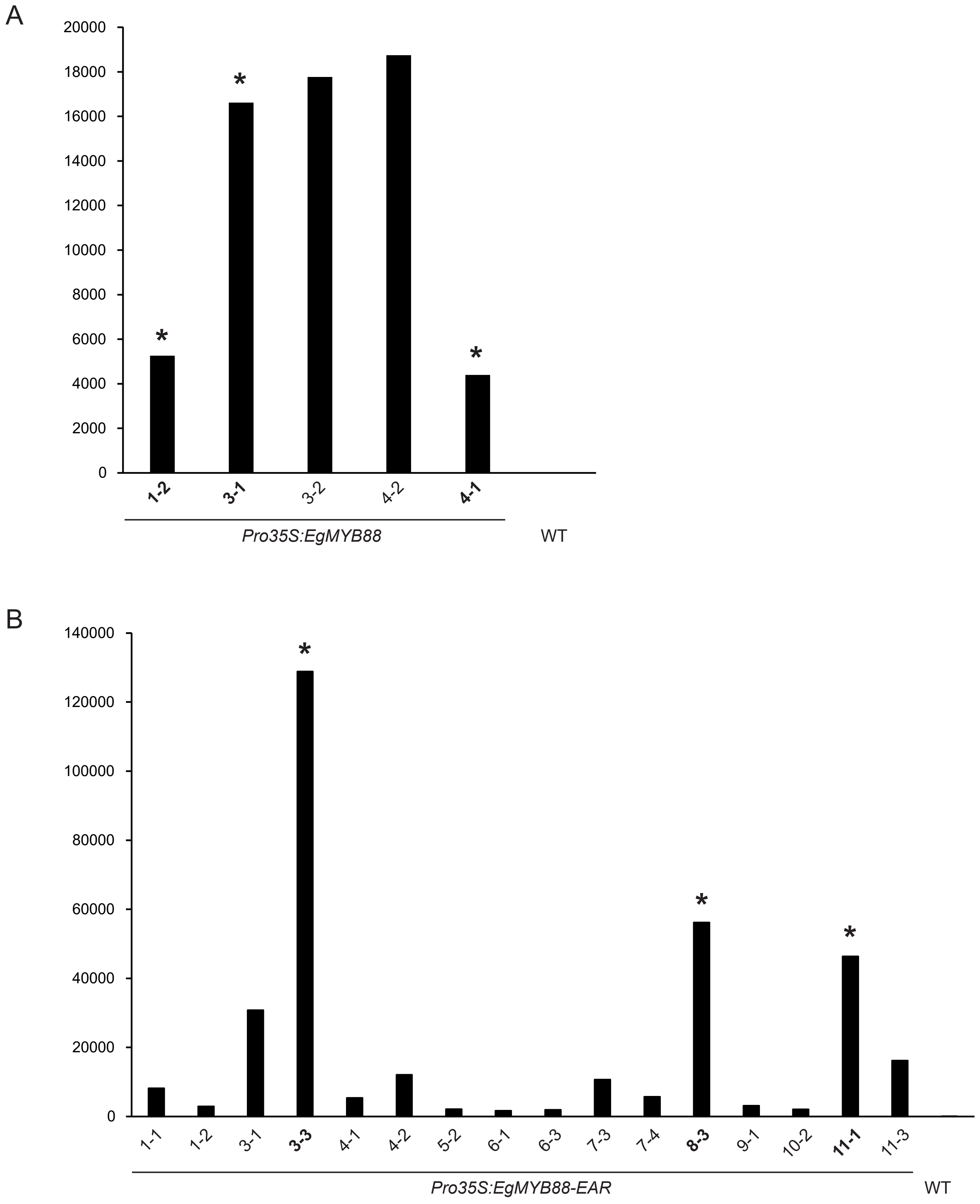

Figure S2. EgMYB88 transgene expression levels in several lines of *EgMYB88* (A) and *EgMYB88-EAR* (B) transformed poplar plants. Selected lines for detailed characterization of their phenotypes are highlighted in bold and with an asterisk.

Figure S3

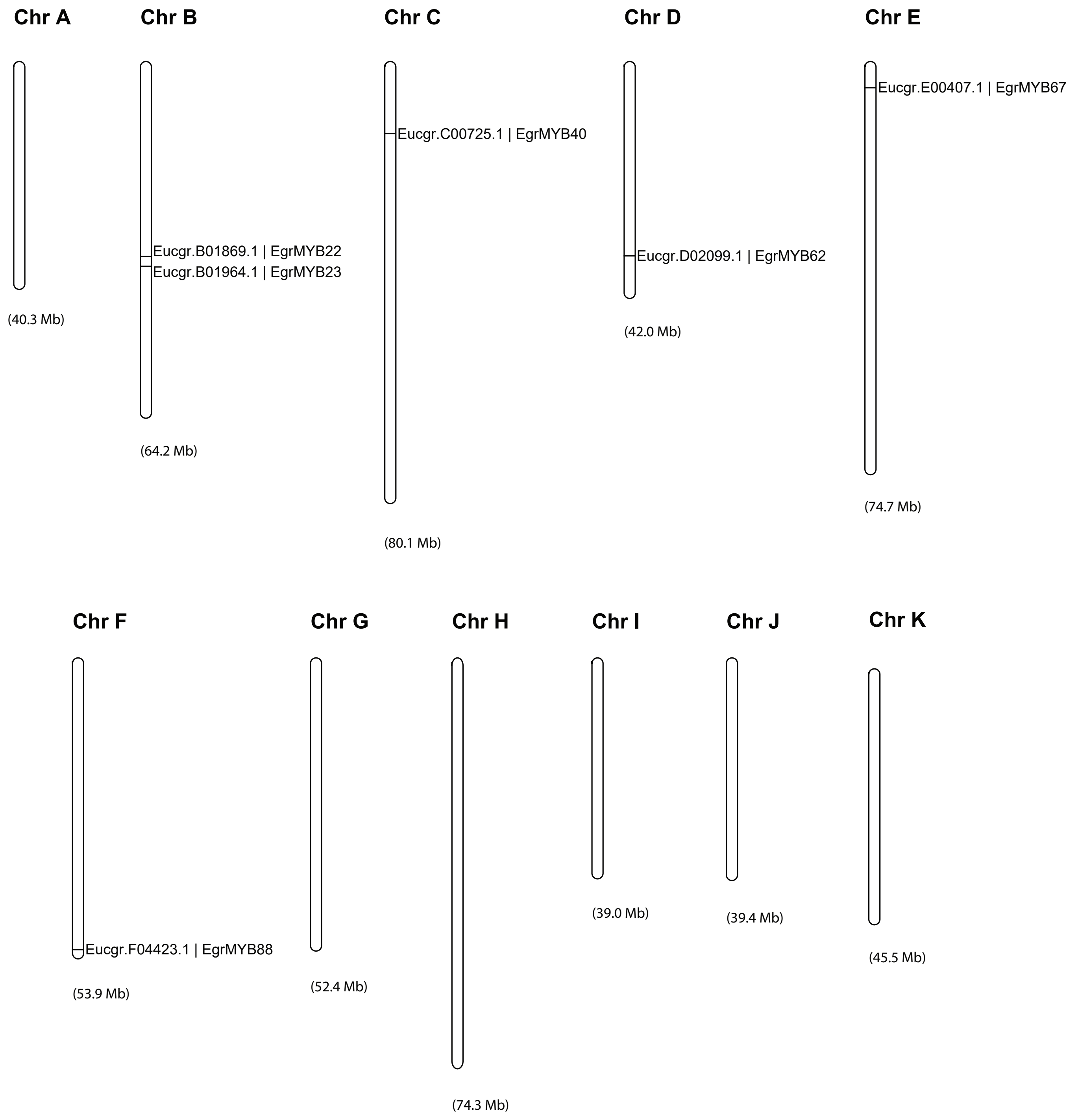

Figure S3. Physical position of the six genes from WPS-I on the 11 *E. grandis* chromosomes. Data about physical position was obtained from Soler et al. (2015).

Figure S4

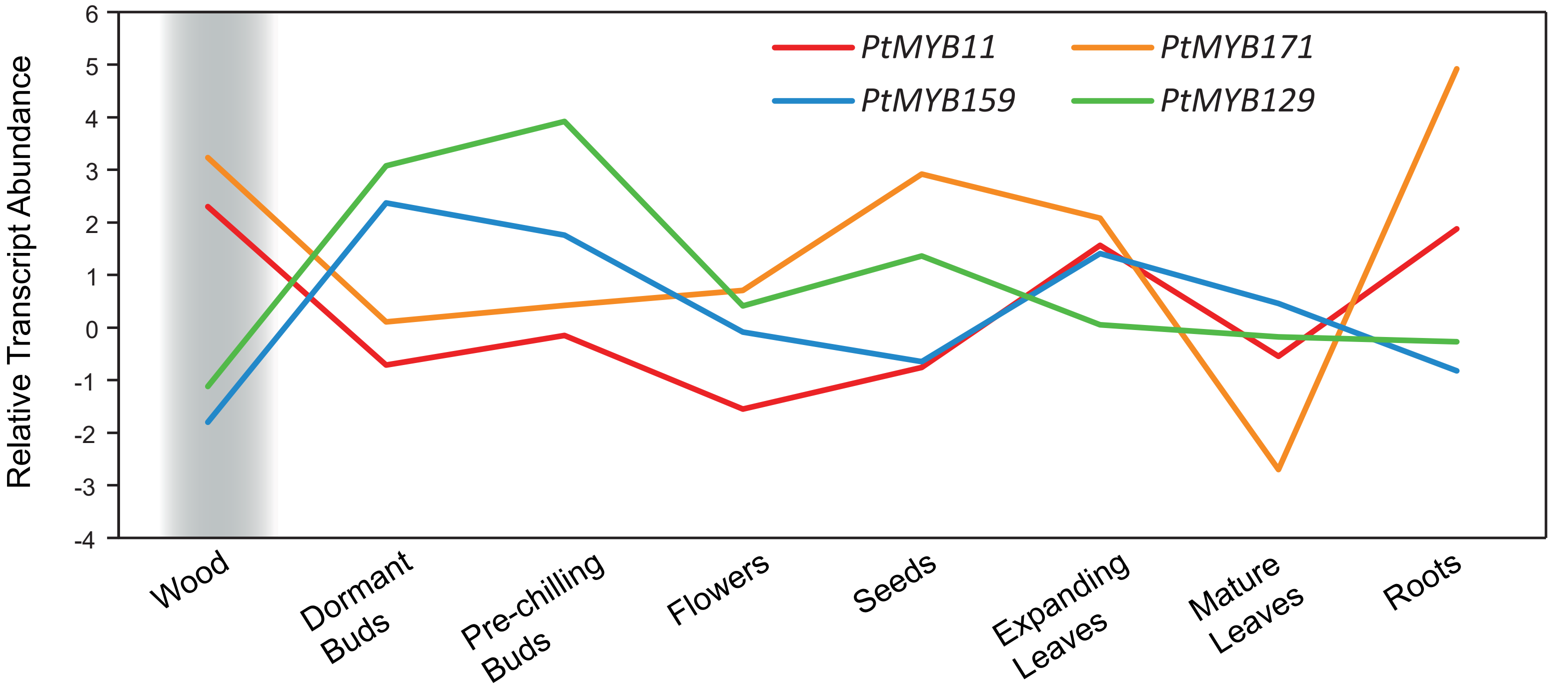

Figure S4. RNAseq expression values from the different poplar genes from WPS-I, extracted from PopGenIE (Sjödin et al., 2009) as part of an expression atlas of *P. tremula* (Sundell et al., 2015). Vascular tissue is highlighted with a grey background.

Figure S5

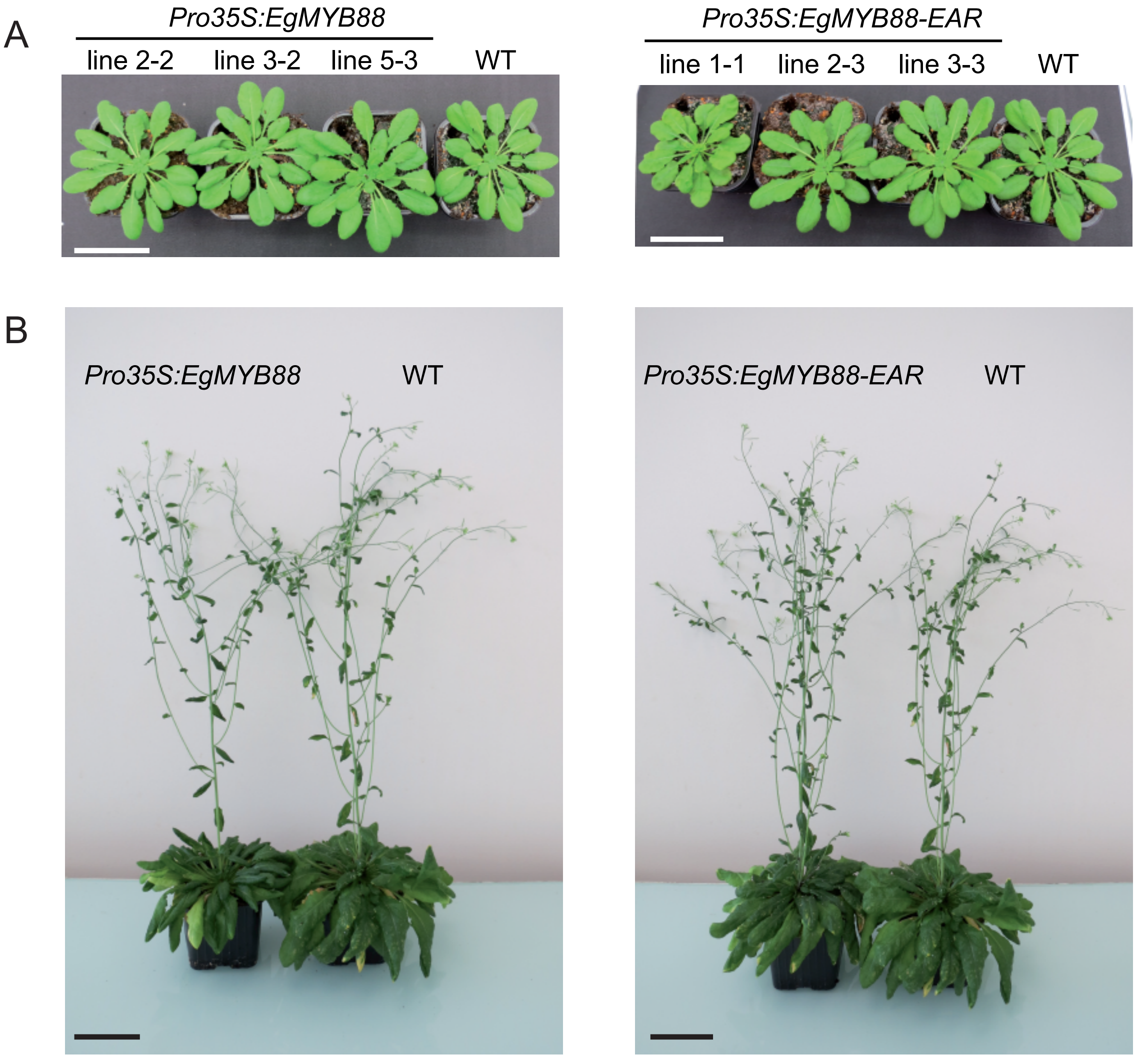

Figure S5. Macroscopic images of transgenic *Arabidopsis* plants grown in short-day conditions for four weeks (A) and until sampling (B). No visible differences were observed compared to control plants. Scale bar, 5 cm.

Figure S6

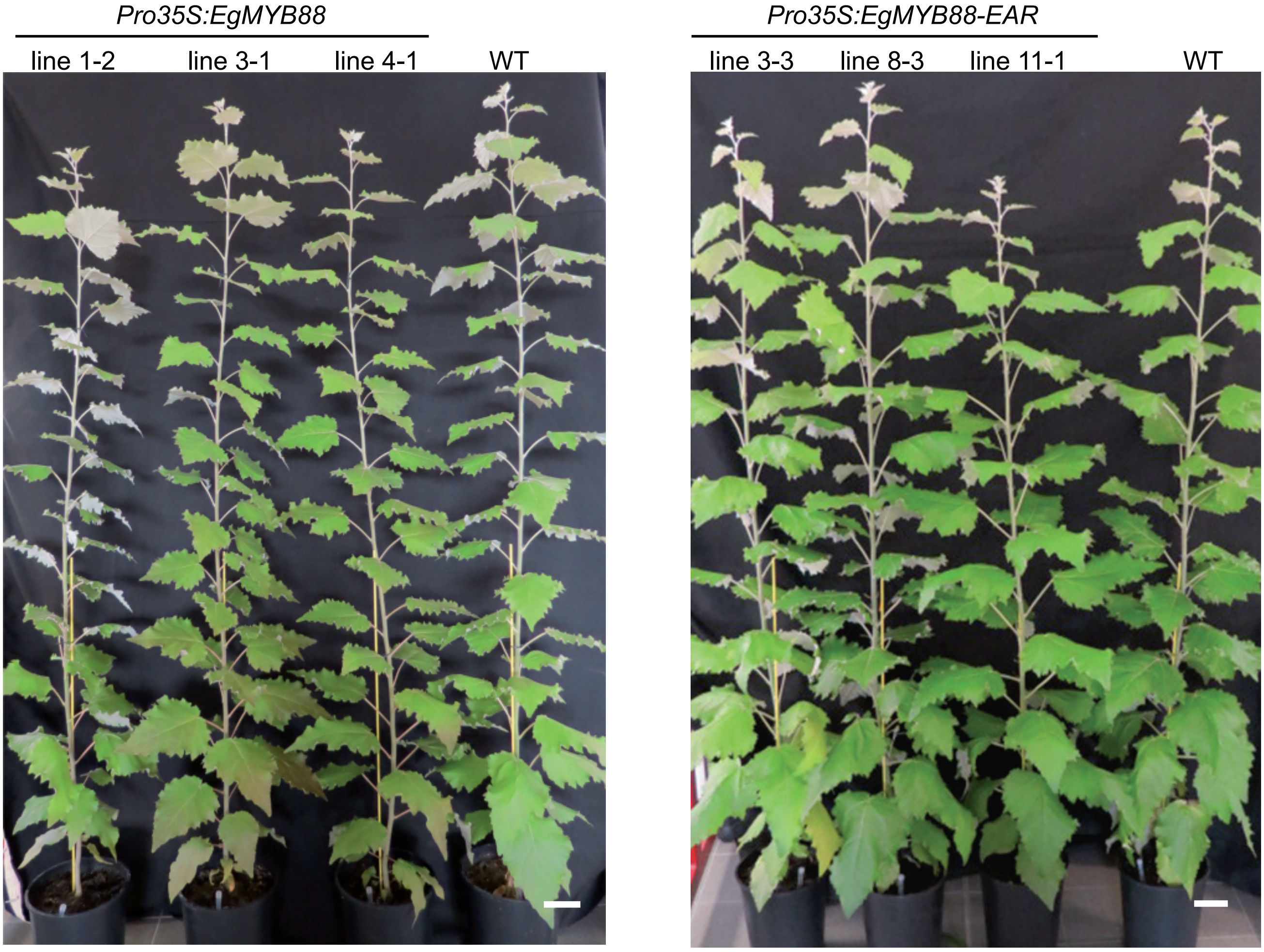

Figure S6. Macroscopic image of transgenic poplar plants just before sampling. No visible differences were observed compared to control plants. Scale bar, 5 cm.

Figure S7

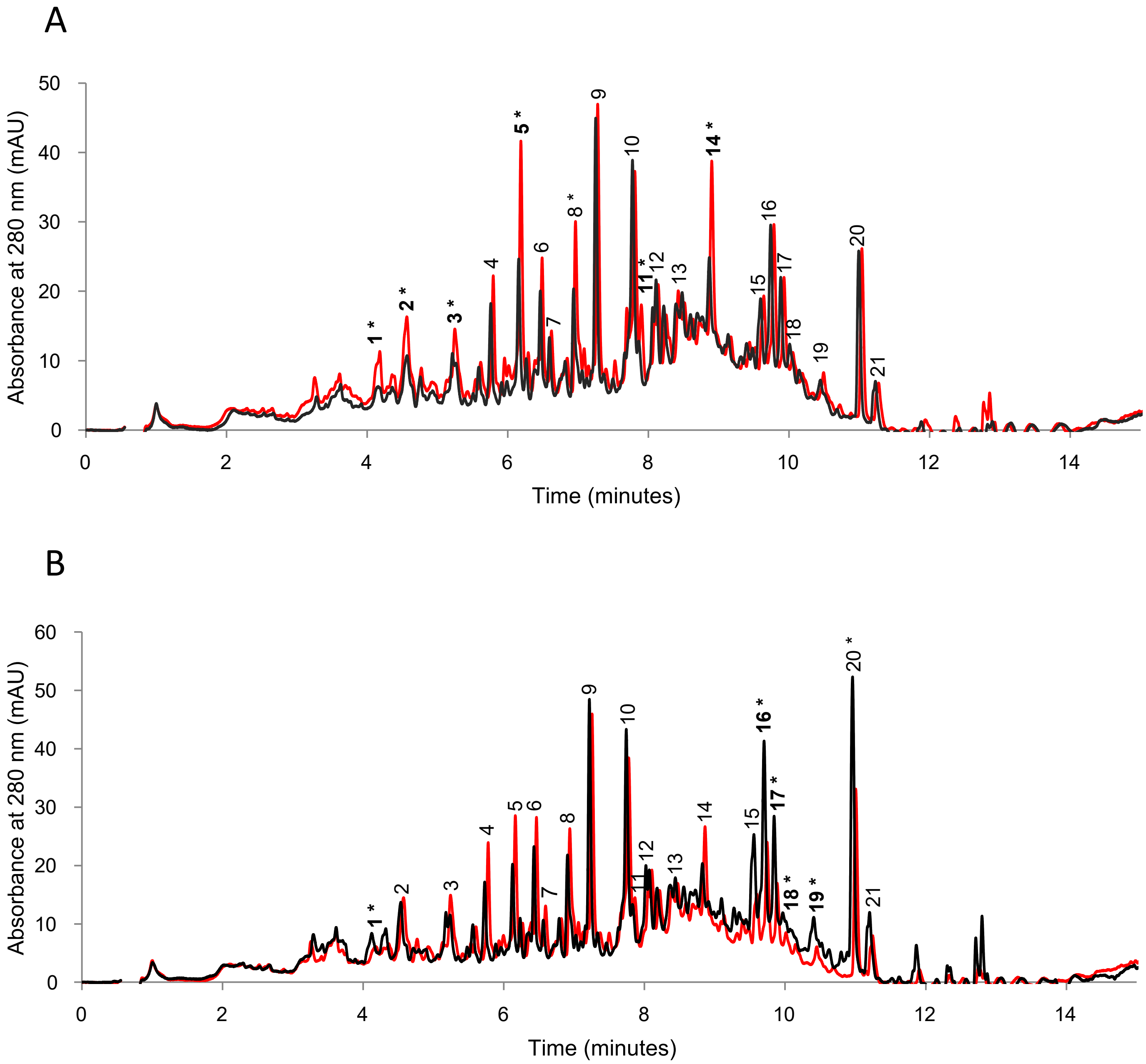

Figure S7. Chromatogram profiles of the water-methanol extracts of poplar *Pro35S:EgMYB88* (A) and *Pro35S:EgMYB88-EAR* (B) with their respective controls. Peak number refers to Table S4 and Table S5. Peaks corresponding to molecules with levels significantly different from controls are highlighted in bold with an asterisk. On each panel, the black curve corresponds to the control plants and the red to the transgenic line.

Table S2. Amino acid similarities between the *E. grandis* and the *P. trichocarpa* R2R3-MYB sequences belonging to WPS-I.

	EgrMYB88	*PtrMYB11*	*PtrMYB171*	*PtrMYB159*	*EgrMYB22*	*EgrMYB23*	*EgrMYB40*	*PtrMYB129*	*EgrMYB62*	*EgrMYB67*
EgrMYB88	100%									
PtrMYB11	**73.00%**	100%								
PtrMYB171	**73.00%**	85.00%	100%							
PtrMYB159	65.48%	66.37%	69.00%	100%						
EgrMYB22	64.60%	66.81%	65.92%	75.00%	100%					
EgrMYB23	63.71%	65.92%	65.04%	74.00%	98.00%	100%				
EgrMYB40	63.71%	64.15%	67.69%	76.00%	86.72%	88.00%	100%			
PtrMYB129	56.63%	57.52%	57.52%	58.40%	57.07%	56.63%	56.00%	100%		
EgrMYB62	65.04%	63.71%	62.83%	69.91%	65.92%	65.04%	64.60%	65.00%	100%	
EgrMYB67	65.04%	61.94%	61.94%	66.81%	64.15%	62.83%	64.60%	66.81%	79.00%	100%

Highlighted in bold there are the highest similarity values between *EgrMYB88* and the poplar sequences within the WPS-I. Alignment was performed using MAFFT with the FFT-NS-i method (Katoh et al. 2002) and similarity results were calculated using the Sequence Identity and Similarity Tool (SIAS, http://imed.med.ucm.es/Tools/sias.html)

Table S3. Relative amounts of the different categories of phenolics compounds identified by Py-GC/MS in non-extracted *Arabidopsis* hypocotyls.

**Arabidopsis lines**	**% Total phenolics**	**% G-type phenolics**	**% S-type phenolics**	**% H-type phenolics**	**% Other phenolics**	**S/G ratio**
Pro35S:EgMYB88	12.53 ± 0.92	60.06 ± 1.41	18.18 ± 2.02	13.34 ± 1.31	8.42 ± 0.23	0.30 ± 0.04
Controls	13.11 ± 2.25	59.20 ± 1.06	19.64 ± 2.18	12.59 ± 2.08	8.56 ± 1.09	0.33 ± 0.04

Data represent the means ± standard deviation of six biological replicates from *Pro35S:EgMYB88* lines and nine biological replicates from control lines (each replicate is constituted by a pool of three hypocotyls from three plants growing at the same time). No statistical differences were found using a Student's t test with a P value < 0.05.

Table S4. Complete metabolic profiling in stems of *Pro35S:EgMYB88* transgenic poplar lines.

**Peak number**	**Retention time (min)**	**UV lambda max**	**M-**	**Fragment MS/MS**	**Formula**	**Compound name**	***Pro35S:EgMYB88*** peak area (x1000)	ratio	**Control** Peak area (x1000)
**1**	**4.154**	**280**	**289.0703**	**245 (100), 205 (40), 179 (20)**	**C15H14O6**	**Catechin**	**80.3 ± 26.8 ***	**2.6**	**30.4 ± 3.1**
**2**	**4.562**	**288, 321**				**Unknown**	**68.7 ± 5.0 ****	**1.4**	**47.5 ± 3.3**
**3**	**5.194**	**280**				**Unknown**	**62.1 ± 7.7 ***	**1.3**	**46.1 ± 6.1**
4	5.765	272				Unknown	48.5 ± 3.7	1.1	44.3 ± 5.5
**5**	**6.160**	**270**	**423.1278**	**155 (100), 137 (90), 317 (40), 299 (40), 123 (40)**	**C20H24O10**	**Salicortin**	**91.7 ± 13.7 ****	**1.7**	**53.8 ± 1.9**
6	6.440	343	207.0655	177 (100), 192 (20)	C11H12O4	Sinapaldehyde	337.0 ± 65.6	1.2	276.4 ± 16.0
7	6.595	281	477			Unknown	28.6 ± 13.8	1.7	16.8 ± 2.6
**8**	**6.929**	**282**	**405.1172**	**283 (100), 121 (20), 163 (2), 181 (1)**	**C20H22O9**	**Salireposide**	**80.3 ± 4.6 *****	**1.6**	**51.6 ± 0.7**
9	7.246	276				Unknown	112.9 ± 5.0	1.1	104.3 ± 4.4
10	7.757	276	583.2172	535 (100), 369 (40)	C31H35O11	G(8-O-4)S(8-5)G	102.4 ± 12.5	0.9	110.3 ± 7.5
**11**	**7.863**	**286, 310**	**435.1283**			**Unknown**	**50.5 ± 7.8 ***	**1.6**	**30.9 ± 8.4**
12	8.107	280, 343	581.2022	533 (100), 367 (90), 355 (40), 551 (40)	C31H33O11	G(8-O-4)S(8-5)G’	102.5 ± 9.4	0.9	108.7 ± 8.7
13	8.354	339	809.3003	809 (100), 595 (40), 369 (1), 761 (1), 613	C42H49O16	G(8-O-4)S(8-O-4)S(8-8)S	39.1 ± 7.4	1.0	39.7 ± 7.5
**14**	**8.835**	**271**	**527.1553**	**405 (100), 155 (15), 299 (10)**	**C27H28O11**	**Tremulacin**	**70.4 ± 11.4 ****	**1.9**	**37.6 ± 2.8**
15	9.635	266				Unknown	33.2 ± 12.3	1.1	29.1 ± 5.7
16	9.708	266	703.2379	703 (100), 655 (6), 561 (3), 503 (2), 369 (2), 667 (1)	C38H39O13	G(8-O-4)SP(8-5)G	60.3 ± 18.9	1.1	55.2 ± 10.8
17	9.852	266	703.238	703 (100), 655 (6), 561 (3), 503 (2), 369 (2), 667 (1)	C38H39O13	G(8-O-4)SP(8-5)G	33.5 ± 9.4	1.1	31.3 ± 6.5
18	9.989	339	701.2224	701 (100), 653 (10), 367 (10) 559 (3), 515 (2), 665 (1)	C38H37O13	G(8-O-4)SP(8-5)G’	22.6 ± 4.0	0.9	24.8 ± 7.7
19	10.110	339	701.2224	701 (100), 653 (10), 367 (10) 559 (3), 515 (2), 665 (1)	C38H37O13	G(8-O-4)SP(8-5)G’	13.6 ± 2.3	1.2	11.8 ± 3.2
20	10.994	326	727.238	727 (100), 551 (1), 501 (1)		Putative oligolignol	93.9 ± 27.0	0.9	104.3 ± 17.4
21	11.234	272, 328	727.238	727 (100), 551 (1), 501 (1)		Putative oligolignol	30.2 ± 9.9	1.0	29.2 ± 5.5

Data represent the means ± standard deviation of three *Pro35S:EgMYB88* poplar (each *Pro35S:EgMYB88* plant belongs to one of the three selected independent lines specified in Figure S2A) and three control plants. Statistics were calculated with Student’s t test, *** P value < 0.001, ** P value < 0.01, * P value < 0.05. Significant differences relative to the control values are highlighted in bold. G(8–O–4)S(8–5)G consists in coniferyl alcohol (8-O-4) sinapyl alcohol (8-5) coniferyl alcohol; G(8–O–4)S(8–5)G' consists in coniferyl alcohol (8-O-4) sinapyl alcohol (8-5) coniferaldehyde; G(8–O–4)G(8–O–4)S(8–8)S consists in coniferyl alcohol (8-O-4) coniferyl alcohol (8-O-4) sinapyl alcohol (8-8) sinapyl alcohol; G(8–O–4)SP(8–5)G consists in coniferyl alcohol (8-O-4) sinapyl p-hydroxybenzoate (8-5) coniferyl alcohol; G(8–O–4)SP(8–5)G' consists in coniferyl alcohol (8-O-4) sinapyl p-hydroxybenzoate (8-5) coniferaldehyde.

Table S5. Complete metabolic profiling in stems of *Pro35S:EgMYB88-EAR* transgenic poplar lines

Peak number	Retention time (min)	UV lambda max	M-	Fragment MS/MS	Formula	Compound name	*Pro35S:EgMYB88-EAR* peak area (x1000)	*Pro35S:EgMYB88-EAR* ratio	Control Peak area (x1000)
**1**	**4.154**	**280**	**289.0703**	**245 (100), 205 (40), 179 (20)**	**C15H14O6**	**Catechin**	**14.2 ± 3.6 ***	**0.6**	**24.5 ± 4.9**
2	4.562	288, 321				Unknown	59.5 ± 7.5	1.0	56.9 ± 9.8
3	5.194	280				Unknown	57.1 ± 1.5	1.2	46.3 ± 7.6
4	5.765	272				Unknown	45.6 ± 10.2	0.8	59.8 ± 13.2
5	6.160	270	423.1278	155 (100), 137 (90), 317 (40), 299 (40), 123 (40)	C20H24O10	Salicortin	52.7 ± 13.9	0.9	59.6 ± 16.6
6	6.440	343	207.0655	177 (100), 192 (20)	C11H12O4	Sinapaldehyde	373.3 ± 60.2	1.1	342.9 ± 32.5
7	6.595	281	477			Unknown	22.0 ± 2.3	1.2	18.6 ± 5.6
8	6.929	282	405.1172	283 (100), 121 (20), 163 (2), 181 (1)	C20H22O9	Salireposide	65.4 ± 11.9	0.9	70.5 ± 19.2
9	7.246	276				Unknown	113.3 ± 6.9	1.0	117.0 ± 12.4
10	7.757	276	583.2172	535 (100), 369 (40)	C31H35O11	G(8-O-4)S(8-5)G	108.6 ± 1.3	1.0	111.1 ± 13.6
11	7.863	286, 310	435.1283			Unknown	35.7 ± 5.3	0.9	38.9 ± 8.8
12	8.107	280, 343	581.2022	533 (100), 367 (90), 355 (40), 551 (40)	C31H33O11	G(8-O-4)S(8-5)G’	78.2 ± 3.2	1.1	72.6 ± 19.2
13	8.354	339	809.3003	809 (100), 595 (40), 369 (1), 761 (1), 613	C42H49O16	G(8-O-4)S(8-O-4)S(8-8)S	23.4 ± 1.8	1.0	22.6 ± 4.9
14	8.835	271	527.1553	405 (100), 155 (15), 299 (10)	C27H28O11	Tremulacin	41.9 ± 10.0	0.9	47.1 ± 24.1
**15**	**9.635**	**266**				**Unknown**	**32.3 ± 1.7 ***	**0.7**	**49.0 ± 10.1**
**16**	**9.708**	**266**	**703.2379**	**703 (100), 655 (6), 561 (3), 503 (2), 369 (2), 667 (1)**	**C38H39O13**	**G(8-O-4)SP(8-5)G**	**63.0 ± 8.4 ***	**0.7**	**96.6 ± 15.5**
**17**	**9.852**	**266**	**703.238**	**703 (100), 655 (6), 561 (3), 503 (2), 369 (2), 667 (1)**	**C38H39O13**	**G(8-O-4)SP(8-5)G**	**32.5 ± 2.4 ****	**0.7**	**49.7 ± 6.4**
**18**	**9.989**	**339**	**701.2224**	**701 (100), 653 (10), 367 (10) 559 (3), 515 (2), 665 (1)**	**C38H37O13**	**G(8-O-4)SP(8-5)G’**	**15.0 ± 1.6 ****	**0.6**	**24.6 ± 2.5**
**19**	**10.110**	**339**	**701.2224**	**701 (100), 653 (10), 367 (10) 559 (3), 515 (2), 665 (1)**	**C38H37O13**	**G(8-O-4)SP(8-5)G’**	**8.8 ± 0.8 ****	**0.7**	**12.6 ± 1.3**
**20**	**10.994**	**326**	**727.238**	**727 (100), 551 (1), 501 (1)**		**Putative oligolignol**	**145.7 ± 5.8 ***	**0.7**	**196.3 ± 30.4**
21	11.234	272, 328	727.238	727 (100), 551 (1), 501 (1)		Putative oligolignol	45.1 ± 3.7	0.8	54.0 ± 5.1

Data represent the means ± standard deviation of three *Pro35S:EgMYB88-EAR* poplar (each *Pro35S:EgMYB88-EAR* plant belongs to one of the three selected independent lines specified in Figure S2B) and four control plants. Statistics were calculated with Student’s t test, ** *P* value < 0.01, * *P* value < 0.05. Significant differences relative to the control values are highlighted in bold. G(8–O–4)S(8–5)G consists in coniferyl alcohol (8-O-4) sinapyl alcohol (8-5) coniferyl alcohol; G(8–O–4)S(8–5)G' consists in coniferyl alcohol (8-O-4) sinapyl alcohol (8-5) coniferaldehyde; G(8–O–4)G(8–O–4)S(8–8)S consists in coniferyl alcohol (8-O-4) coniferyl alcohol (8-O-4) sinapyl alcohol (8-8) sinapyl alcohol; G(8–O–4)SP(8–5)G consists in coniferyl alcohol (8-O-4) sinapyl p-hydroxybenzoate (8-5) coniferyl alcohol; G(8–O–4)SP(8–5)G' consists in coniferyl alcohol (8-O-4) sinapyl p-hydroxybenzoate (8-5) coniferaldehyde.
